# Synthesis, Spectroscopic Analysis and Assessment of the Biological Activity of New Hydrazine and Hydrazide Derivatives of 3-Formylchromone

**DOI:** 10.3390/molecules23082067

**Published:** 2018-08-17

**Authors:** Krzysztof Słomiak, Andrzej Łazarenkow, Lilianna Chęcińska, Joachim Kusz, Justyn Ochocki, Jolanta Nawrot-Modranka

**Affiliations:** 1 Department of Bioinorganic Chemistry, Medical University of Lodz, Muszyńskiego 1, 90-151 Łódź, Poland; andrzej.lazarenkow@umed.lodz.pl (A.Ł.); justyn.ochocki@umed.lodz.pl (J.O.); jolanta.nawrot-modranka@umed.lodz.pl (J.N.-M.); 2 Department of Physical Chemistry, Faculty of Chemistry, University of Lodz, Pomorska 163/165, 90-236 Łódź, Poland; lilianna.checinska@chemia.uni.lodz.pl; 3 Department of Physics of Crystals, Institute of Physics, University of Silesia, Uniwersytecka 4, 40-007 Katowice, Poland; joachim.kusz@us.edu.pl

**Keywords:** hydrazine, hydrazide, benzo-γ-pyrones, 3-formylchromone, XTT-assay, anti-proliferative activity

## Abstract

The hydrazine and hydrazide derivatives of benzo-γ-pyrones with fluorine substituents remain an unexplored group of chemical compounds. This preliminary study reports the synthesis, structural assessment, initial microbiological screening and biological testing of the synthesized compounds on cell lines using the XTT-assay. A series of 10 novel hydrazine and hydrazide derivatives of 3-formylchromone were synthesized and their structures determined. Structural assessment consisted of elemental analysis, IR, ^1^H-NMR, ^13^C-NMR, MS and crystallographic studies. Antimicrobial activity was tested on standard strains representing different groups of microorganisms. The tested compounds were found to inhibit microbial growth. Concentrations of 0.01–1250 µmol/L were found to influence cell proliferation, demonstrating antiproliferative and stimulation of proliferation against two cell lines: the L929 cell line (mouse fibroblast cell line) and the EA.hy926 cell line (the human umbilical vein, somatic cell hybrid).

## 1. Introduction

Due to the increasing problem of drug resistance, the search for novel and efficient antimicrobial and antiproliferative agents is a worldwide concern. Benzopyrones occur widely in Nature, and benzopyrone derivatives have been reported in algae [1], fungi [2,3] and plants [4,5] including trees [6,7]. These compounds show broad pharmacological activities, and studies have found them to have antimicrobial, antitumor, antioxidant, anti-HIV and antiallergenic properties [8,9,10,11,12,13,14]. They have also been suggested as playing a potentially important role in the development of Alzheimer’s Disease treatment [15].

3-Formylchromone is a benzopyrone derivative characterized by high chemical reactivity, especially when nitrogen nucleophilic groups are present. Its structure includes three electron-deficient sites (C–2, C–4, –CHO) where attack by strong nucleophilic agents results in the production of various types of compounds [16]. However, no procedure resulting in the formation of hydrazine and hydrazide derivatives of 3-formylchromone by spontaneous, internal rearrangement of the benzo-γ-pyrone ring has been described [17,18,19,20]. Due to its many possible chemical modifications, the benzo-γ-pyrone structure offers a broad scope of possibilities for the search of the new pharmaceuticals. Previous studies suggest that chemical synthesis of these new compounds is possible [21].

The effects of fluorine substituents on the bioactivity of existing compounds may often be poorly understood and unpredictable, but they are nevertheless significant [22]. Fluorinated drugs are used for treatment of diseases of the central nervous system, cardiovascular diseases and obesity, as well as in antibacterial and antifungal therapy [23]. The presence of a fluorine substituent in a molecule can influence its conformation, pK_a_, intrinsic potency, membrane permeability, metabolic pathways and pharmacokinetic properties [24]. Fluoride itself is mostly used as an anticaries agent on a daily basis. It is known to have an important effect on the bacteria of dental plaque. Its action affects bacterial metabolism as an enzyme inhibitor [25,26]. Hence the presence of a fluorine substituent can play a major role on the usability of a compound as a novel treatment alternative.

Copper plays important metabolic role in living organisms. Copper action is observed in redox chemistry of mitochondrial respiration and physiological stability maintenance by free radicals elimination as well as iron absorption [27,28]. Raised or lowered levels or copper are found in many diseases such as rheumatoid arthritis, gastrointestinal ulceration, epilepsy, diabetes and cancer [29]. Locally raised levels of copper are crucial for angiogenesis to occur [30].

Previous studies have confirmed the antiproliferative properties [12,31] and biochemical activity [32,33] of 3-formylchromone derivatives. Moreover, biological activity of copper (II) complexes of 3-formylchromone, was also described [21]; however, hydrazine and hydrazide derivatives of benzopyrones with fluorine substituents remain a poorly-analyzed group of compounds. Therefore, the present study describes the synthesis and subsequent analysis of compounds of this type, with the aim of better understanding their potential biological properties.

The study presents the molecular and crystal structures of synthesized compounds. Initial antimicrobial activity testing was done on standard strains representing different groups of microorganisms. This study also investigates the influence on proliferation on two nonpathogenic cell lines: L929 (mouse fibroblast cell line) and the human umbilical vein somatic cell hybrid EA.hy926.

## 2. Results and Discussion

### 2.1. Chemistry

**Scheme 1:**
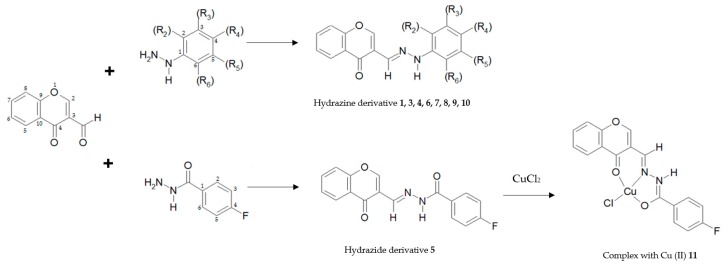
Synthesis of hydrazine and hydrazide derivatives of 3-formylchromone. Reaction conditions: solvent: CH_3_COOC_2_H_5_, time: 48h, temperature: reflux temperature, crystallization from 50:50 acetone/ethanol. Hydrazine derivatives of 3-formylchromone: **1**: R_2_=R_5_=F, R_3_=R_4_=R_6_=H; **3**: R_4_=CF_3_, R_2_=R_3_=R_5_=R_6_=H; **4**: R_3_=R_5_=CF_3_, R_2_=R_4_=R_6_=H; **6**: R_2_=R_3_=R_5_=R_6_=F, R_4_=H; **7**: R_2_=CH_3_, R_5_=F, R_3_=R_4_=R_6_=H; **8**: R_2_=R_4_=R_6_=F, R_3_=R_5_=H; **9**: R_2_=R_3_=R_4_=R_5_=R_6_= F, **10**: R_2_=CF_3_, R_3_=R_4_=R_5_=R_6_=H.

3-Formylchromone was previously synthesized in the Department of Bioinorganic Chemistry, Medical University of Lodz using the Vilsmeier-Haack reaction [34] with use of *o*-hydroxyacetophenone and DMF/POCl_3_ acting as substrate and solvent, respectively. The synthesis of novel hydrazine (compounds **1**, **3**, **4**, **6**, **7**, **8**, **9**, **10**) and hydrazide (compounds **5**, **11**) derivatives of benzo-γ-pyrones with fluorine substituents by the condensation reaction with 3-formylchromone is shown in Scheme 1. All reactions were carried out at reflux temperature because they did not proceed at room temperature. The syntheses afford moderate yields (60–80%). The structures of all compounds were established and confirmed by ^1^H-NMR, ^13^C-NMR, IR, MS and elemental analysis. All the compounds present similar spectroscopic characteristics because of their structural similarity. In the IR spectra two groups are considered to be present and characteristic for all ligands and complexes: 1620–1644 cm^−1^ (C=O) and 1517–1584 cm^−1^ (CH=N). Compounds **1**, **3**, **5**, **8**, **10** were synthesized in the form of crystals and for them X-ray diffraction studies were performed.

### 2.2. X-ray Diffraction Studies

**Figure 1:**
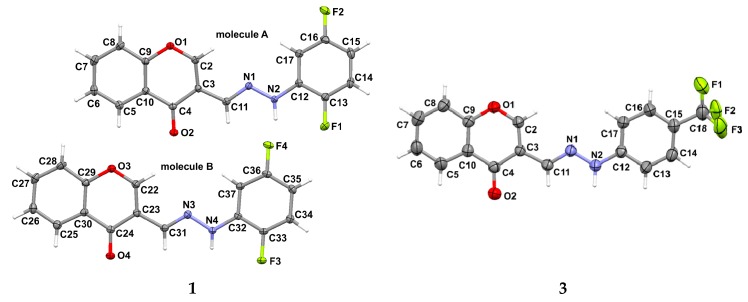

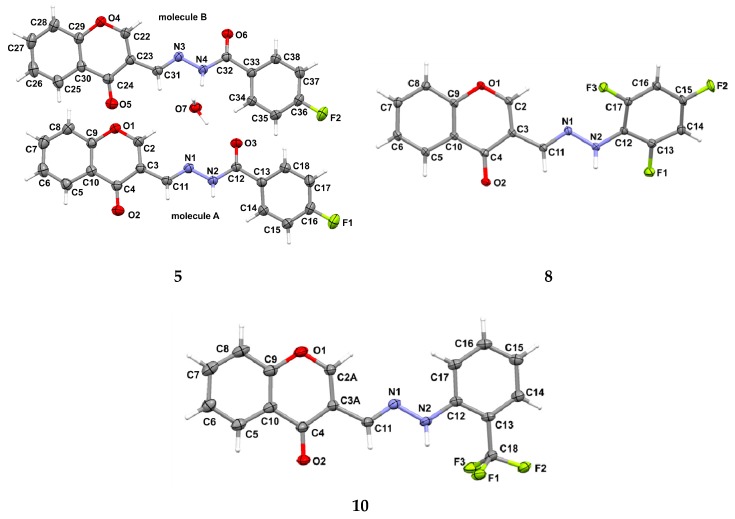
Molecular structures of crystal compounds with anisotropic displacement ellipsoids drawn at the 50% (**1**, **8**, **10**) and 30% (**3**, **5**) probability level with atomiC-Numbering scheme. Hydrogen atoms are shown as spheres of arbitrary radius. For clarity, only three from six fluorine atoms (F1, F2, F3) with occupancy factor equal to 0.5 are shown for structure **3**. For structure **10**, the carbon atoms with higher site occupancy factors are shown.

The molecular structures of **1**, **3**, **5**, **8**, **10** with their atomic labeling and anisotropic displacement ellipsoids are depicted in Figure 1. Compounds **1** and **5** crystallize with two molecules in the asymmetric unit. Structure **5** crystallizes as a hydrate. All non-hydrogen atoms were refined anisotropically. The hydrogen atoms, except those attached to the nitrogen atom, H2A and H4A; and the hydrogen atoms of the water molecule, were geometrically fixed at calculated positions using a riding model.

In **3**, the fluorine atoms in the CF_3_ group were refined as disordered over two equally-occupied positions each, and their anisotropic displacement parameters (*U*_ij_) were restrained to be similar. Additionally, geometrical similarity constraints were applied to all C–F bond lengths by SADI instruction.

In **10,** two carbon atoms from the heterocyclic ring (C2 and C3) were found to be disordered and refined with two alternative positions with the final site-occupancy factors A to B components: *k*_A_:*k*_B_ = 0.856 (5):0.144 (5). Crystallographic data and the final figures of merit for structure refinements are summarized in Table 1.

The examined molecules consist of a 4*H*-chromene-4-one system (a heterocyclic ring fused with a benzene ring) substituted at position *3* by a phenylhydrazonomethyl group, with a methylene-benzohydrazide group being present in structure **5**. In all structures, the hydrazine moiety is oriented similarly with respect to the chromone ring. The structures differ from each other in fluorine substituents. **1** and **8** differ only in the number of fluorine atoms substituted at the phenyl ring: two and three atoms, respectively. Both crystallize in a triclinic system, in the *P*ī space group; however, two independent molecules were identified in an asymmetric unit for compound **1**. Compound **5** also crystallizes with two molecules in an asymmetric unit, but only one fluorine atom is attached to the terminal phenyl ring at the *para* position to the hydrazide group. The trifluoromethyl group is characteristic of structures **3** and **10**, which are substituted at positions 4 and 2 of the terminal phenyl group, respectively. Selected geometrical parameters are listed in Table S1 (see Supplementary Materials). The bond lengths and valence angles of the studied structures are generally similar; however, the bonds in **3** and **5** seem to be slightly shorter, probably due to the X-ray measurement at room temperature. On the other hand, in **10**, the disorder of two carbon atoms, C2 and C3, within the heterocyclic ring results in greater discrepancies in the geometrical parameters observed within the disordered region in comparison to the other compounds. In contrast to bond lengths and angles, some torsion angles differ quite significantly, indicating changes in the molecular conformations of examined structures. The heterocyclic ring is planar, and together with a flat benzene ring, forms a chromone ring, which can be also considered as substantially planar. In seven molecules analyzed, the total puckering amplitude (Q) for the 10-membered ring was found to range from 0.028 (2) Å to 0.069 (3) Å [35], for **5** (molecule B) and **5** (molecule A), respectively. Furthermore, the maximum deviation of carbon atom C4 from the best plane of chromone moiety is 0.045 (2) Å, observed in **5** (molecule A).

**Figure 2:**
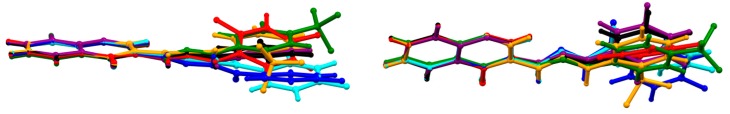
Superimposed structures: molecule A of **1** (purple), molecule B of **1** (black), **3** (green), molecule A of **5** (cyan), molecule B of **5** (blue), **8** (red) and **10** (orange).

The spatial arrangement of two planar fragments, i.e., the 10-membered chromone ring and the terminal phenyl group, is shown in Figure 2. Superimposing the structures of the seven considered molecules, it can be seen that the dihedral angle between the best planes of the chromone and phenyl rings differ quite significantly from 2.96 (6)° in **10** (indicated in orange in Figure 2) to 25.88 (3)° in **8** (indicated in red in Figure 2). Since compound **5** has an additional carbonyl group between the hydrazine and phenyl fragments, its independent molecules (cyan and blue in Figure 2) demonstrate conformations which are considerably different to other derivatives, and even to each other. In **5** two independent molecules differ mostly within carbonyl group and attached phenyl ring, thus the dihedral angle between the best planes of terminal phenyl ring and the 10-membered ring is 12.48 (10)° and 7.82 (10)° for molecules A and B, respectively. For comparison, in **1** the corresponding dihedral angle is 12.75 (6)° and 4.68 (6)°, for molecules A and B, respectively.

**Figure 3:**
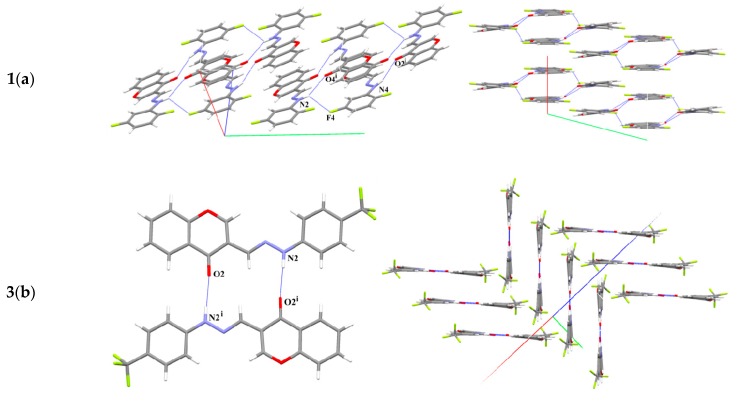

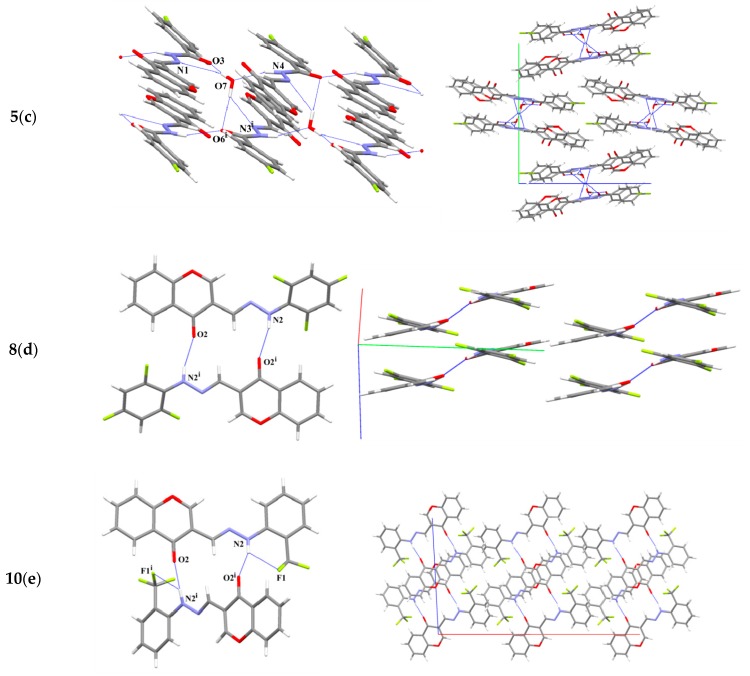
A part of the studied crystal structures showing characteristic hydrogen-bonded motifs (**left**) and their arrangement in the crystal lattices (**right**) of **1**(**a**), **3**(**b**), **5**(**c**), **8**(**d**) and **10**(**e**). Symmetry codes: under Table 2.

The similarities and differences in the molecular structures of the studied compounds are reflected in their crystal packing arrangements, as illustrated in Figure 3. Table 2 lists some important hydrogen bonding geometries in the analyzed structures.

In **1**, symmetrically-related molecules A and B form dimers through two N–H⋅⋅⋅O hydrogen bonds; there is no pseudo-symmetry between monomers. Rather weak N2–H2A⋅⋅⋅F4 interactions (observed between independent molecules) links dimers in a tetramer (Figure 3a). The inversion centers enable stacking π⋅⋅⋅π interactions. The molecular stacks are built from molecules that are centrosymmetrically oriented along the crystallographic *a* direction, each stack being composed of one type of independent molecules A and B, respectively.

Structure **8**, a related structure, exhibits quite similar packing; however, its higher monoclinic symmetry and the presence of only one independent molecule in the asymmetric unit result in remarkable differences being seen in respect to structure **1**. 

Figure 3 shows the centrosymmetric dimers and their arrangement in the crystal lattice. It can be seen that the molecular conformation of **8** differs significantly from two molecules of **1** (Figure 3a or Figure 3d), thus justifying changes in lattice symmetries and the number of independent molecules in the asymmetric unit.

Centrosymmetric dimers, a characteristic hydrogen-bonded motif, are also found in **3** and **10**. The position of the trifluoromethyl group influences the mutual arrangement of molecules in dimers. The molecules in dimers of **3** (Figure 3b) are almost coplanarly oriented, in contrast to the tightly twisted molecules in dimers of **10**, where the dihedral angle between corresponding chromone rings in dimers is 52 (1)° (Figure 3e).

Since **5** exceptionally crystallizes as hydrate, and its molecular structure demonstrates remarkable differences to those of other analyzed derivatives, it possesses a completely different setup of intermolecular interactions (Table 2, Figure 3c). The molecules are organized to form infinite hydrogen-bonded chains. Figure 3c demonstrates that chain motifs are propagated along the [100] direction.

Furthermore, X-ray crystal determinations have revealed the presence of aromatic π⋅⋅⋅π interactions in four of the five crystal structures investigated. Unexpectedly, in **3**, although most planar hydrogen-bonded dimers are formed with almost linear N–H⋅⋅⋅O interaction between interacting molecules, no stacking interactions are observed. In Figure 3b, it can be clearly seen that the arrangement of dimers it is not conductive to creating π⋅⋅⋅π stacks. In all structures apart from **3**, the distance between ring centroids *Cg*⋅⋅⋅*Cg* ranges from 3.415 (1) Å to 3.921 (1) Å, and the interplanar spacing from 3.269 (1) Å to 3.552 (1) Å. Depending on the structure, different aromatic rings interact with each other (see Table S2 in Supplementary Materials).

### 2.3. Initial Determining the Sensitivity of Microorganisms to the Obtained Chemical Compounds

Fifteen strains of microorganisms from different species were used. The growth inhibition zones were evaluated in two independent experiments (*n* = 2 in each of them), and the potential inhibitors of microbial growth are presented in Table 3. The hydrazine and hydrazide derivatives of 3-formylchromone constitute a class of potential antimicrobial agents and are worthy of further investigation. **1**, **4**, **7**, **10**, **11** exhibit antimicrobial potential at concentrations of 100, 200 µg/mL or at both used concentrations. In case of *N. meningitidis* (result with *) the action of **9** was noted just for 100 µg/mL and the influence in concentration of 200 µg/mL was not observed. The rest of the tested compounds (**3**, **5**, **6**, **8**) were not able to inhibit microbial growth during the experiment and only positive results were presented in Table 3.

### 2.4. Cell Proliferation Study

Four independent experiments (n = 4 for each used concentration) were performed. Cell proliferation study results are presented in Table 4. With regard to their effect on the proliferation of cell lines L929 and EA.hy926, it was found that the IC_50_ values tended to be lower for the EA.hy926 line, except for compound **7** and cisplatin. This indicates good or very good (in the case of **1**, **3**, **5**, **8**, **11**) selectivity of action. The anti-proliferative properties of the studied compounds increased with their concentration. Clear anti-proliferative properties were noted for **5**, **8** and **11**, which demonstrated better performance than cisplatin on both lines (apart from **8** for L929), whereas **1** had such action on EA.hy926 line. Complex **11** was found to have more clearly marked antiproliferative properties than ligand **5**, with its IC_50_ value being 46.89% lower for the L929 and 77.78% lower for EA.hy926. Compounds **1** and **4**, also demonstrated a weak stimulating effect on the proliferation of both cell lines, while compounds **3** and **7** slightly stimulated the proliferation of the EA.hy926 line. Due to the dual action of these compounds, it would be advisable to study their effect on stimulation of proliferation at concentrations below 0.01 μmol/L. This dual action may be desirable during therapy, because the dosage may affect the nature of the compounds.

### 2.5. Statistical Analysis of Cell Proliferation Results

It was impossible to test the statistical normality in the case of **11** (EA.hy926) at 1250 and 500 μM due to n = 4 = *const*. (A = 4 × 0.092 and 4 × 0.069 for 1250 and 500 μM, respectively). At 250 μM, A = 0.066 and 3 × 0.064 were obtained, resulting in *p* = 0.00124 (Shapiro-Wilk test). For **10** (L929) at 0.01 μM, A = 0.061, 0.304, 0.308 and 0.334 were obtained, resulting in *p* = 0.05104 (*t*-test) and 0.02780 (Shapiro-Wilk test).

The rest results of the *t*-test of the cell proliferation study showed that it was not necessary to discard H_0_, because of *p* ≤ 0.01904. The Shapiro-Wilk test found that the following samples met the normal distribution criteria, except: **1** (L929) at 1000 and 750 µM (*p* = 0.00683 and 0.01373, respectively), **1** (EA.hy926) at 250 µM (*p* = 0.01353), **3** (EA.hy926) at 500 and 100 µM (*p* = 0.01684 and 0.00680, respectively), **4** (L929) at Control and 500 µM (*p* = 0.01488 and 0.01513, respectively), **4** (EA.hy926) at 0.1 µM (*p* = 0.01724), **5** (L929) at 1250 µM (*p* = 0.01662), **5** (EA.hy926) at 1 µM (*p* = 0.01843), **6** (L929) at 1250 µM (*p* = 0.01873), **8** (L929) at 0.1 µM (*p* = 0.01476), **8** (EA.hy926) at 1 µM (*p* = 0.02419), **9** (EA.hy926) at 500 µM (*p* = 0.02386), **10** (EA.hy926) at 0.01 µM (*p* = 0.01926). The described incompatibilities were caused by the presence of one outlier result in the series of four experiments.

## 3. Experimental Section

### 3.1. General Information

All hydrazines and hydrazides used for synthesis were purchased from Sigma-Aldrich (Poznan, Poland) and Alfa Aesar (Karlsruhe, Germany) and used without additional purification. Melting points were determined with a use of HMK 73/4521 apparatus (VEB Analytik, Dresden, Germany). The ^1^H-NMR spectra were recorded using an Avance III 600 MHz spectrometer (Bruker, Billerica, MA, USA) with DMSO-*d*_6_ as solvent (measurement frequency: 600 MHz). The ^13^C-NMR spectra were recorded using a Avance III 600 MHz spectrometer (Bruker) with use of DMSO-*d*_6_ (measurement frequency: 150 MHz). IR spectra were recorded using an ATI Mattson Infinity Series FTIR (ATI, Markham, ON, Canada) in KBr. Elemental analysis were performed with a Perkin Elmer 2400 analyzer (Waltham, MA, USA). The mass spectra were recorded using a Synapt G2-Si spectrometer (Waters, Warsaw, Poland) equipped with an electrospray source. ESI-MS spectra were registered in a positive ion mode. The sample was pre-dissolved in 100 μL of THF and then added to 900 μL of methanol.

### 3.2. Synthesis of Hydrazine and Hydrazide Derivatives of 3-Formylchromone

3-Formylchromone (50 mg, 0.287 mmol) was dissolved in ethyl acetate (30 mL) at room temperature. The temperature was raised to reflux temperature and mixture was stirred until the 3-formylchromone was fully dissolved. After 5 min, a suitable amount (1 molar equivalent) of substituted aromatic hydrazine or hydrazide was slowly added (dissolved just before addition in ethyl acetate). Substances were mixed and heated under reflux for 48 h. The obtained precipitates were filtered and air-dried. Resulting substances was recrystallized from 50:50 acetone/ethanol.

### 3.3. Synthesis of Cu(II) 3-Formylchromone Hydrazide Complex

4-Fluorobenzoic acid [1-(4-oxo-4*H*-chromen-3-yl)-meth-(*E*)-ylidene]-hydrazide (50 mg, 0.161 mmol) was dissolved in ethyl acetate (30 mL). The temperature was raised to reflux temperature and mixture was stirred until dissolved. After 5 min, a suitable amount (1 molar equivalent) of anhydrous CuCl_2_ was added. The substances were mixed and heated under reflux for 48 h. The obtained precipitate was filtered and air-dried. The resulting substance was recrystallized from 50:50 acetone/ethanol.

### 3.4. Spectral and Elemental Analysis

*3-[(2,5-Difluorophenyl)-hydrazonomethyl]-chromen-4-one* (**1**). Yellow solid. Yield: 68%. m.p. 226–227 °C. Anal. calcd. for C_16_H_10_F_2_N_2_O_2_: C, 64.00; H, 3.36; N, 9.33. Found: C, 63.95; H, 2.93; N, 9.36%. FT IR (KBr, cm^−1^) ν_max_: 3271.86 (NH_2_), 1628.54 (C=O), 1582.35 (CH=N), 1290.34 (C-O). ^1^H-NMR δ: 10.60 (s, 1H, NH), 8.97 (s, 1H, -CH=), 8.30 (s, 1H, H-2, chromone ring), 8.14–8.15 (dd, *J* = 1.9 and 8.1 Hz, 1H, H-5, chromone ring), 7.84–7.87 (m, 1H, H-7, chromone ring), 7.73–7.74 (d, *J* = 8.3 Hz, 1H, H-8, chromone ring), 7.54–7.57 (m, 1H, H-6, chromone ring), 7.34–7.37 (m, 1H, H-3, phenyl), 7.16–7.20 (m, 1H, H-6, phenyl), 6.53–6.57 (m, 1H, H-4, phenyl). ^13^C-NMR δ: 175.4 (C=O), 160.6 (Ar-CF), 159.0 (Cq), 156.2 (CH), 154.0 (Ar-CF), 146.5 (CH), 144.9 (Ar-Cq), 134.8 (=CH-), 132.9 (CH), 126.3 (Cq), 125.7 (CH), 123.8 (CH), 119.4 (Cq), 116.4 (Ar-CH), 104.4 (Ar-CH), 101.2 (Ar-CH). HRMS (ESI) *m*/*z* calcd. for C_16_H_11_F_2_N_2_O_2_ (M + H)^+^: 301.0789, found 301.0792.

*3-[(4-Trifluoromethylphenyl)-hydrazonomethyl]-chromen-4-one* (**3**). Yellow solid. Yield: 76%. m.p. 215–216 °C. Anal. calcd. for C_17_H_11_F_3_N_2_O_2_: C, 61.45; H, 3.34; N, 8.43. Found: C, 61.02; H, 2.88; N, 8.43%. FT IR (KBr, cm^−1^) ν_max_: 3271.86 (NH_2_), 1628.54 (C=O), 1582.35 (CH=N), 1290.34 (C-O). ^1^H-NMR δ: 10.91 (s, 1H, NH), 8.89 (s, 1H, -CH=), 8.15–8.16 (dd, *J* = 1.5 and 7.9 Hz, 1H, H-5, chromone ring), 8.09 (s, 1H, H-2, chromone ring) 7.85–7.88, (m, 1H, H-7, chromone ring), 7.73–7.74 (d, *J* = 8.6 Hz, 1H, H-8, chromone ring), 7.55–7.57 (m, 3H, H-6, chromone ring, H-2, H-6, phenyl), 7.20–7.22 (d, *J* = 8.7 Hz, 2H, H-3, phenyl; H-5, phenyl). ^13^C-NMR δ: 199.4 (-CF_3_), 175.4 (C=O), 160.4 (Ar-Cq), 160.2 (Ar-Cq), 159.8 (Ar-CH), 159.4 (Cq), 156.2 (CH), 153.5 (Ar-CH), 134.9 (=CH-), 131.2 (CH), 126.6 (Cq), 125.7 (CH), 123.7 (CH), 119.6 (Cq), 119.1 (CH), 112.2 (Ar-CH). HRMS (ESI) *m*/*z* calcd. for C_17_H_12_F_3_N_2_O_2_ (M + H)^+^: 333.0851, found 333.0858.

*3-[(3,5-Bis-trifluoromethylphenyl)-hydrazonomethyl]-chromen-4-one* (**4**). Light yellow solid. Yield: 60%. m.p. 253–254 °C. Anal. calcd. for C_18_H_10_F_6_N_2_O_2_: C, 51.01; H, 2.52; N. 7.00. Found: C, 51.84; H, 1.98; N, 7.07%. FT IR (KBr, cm^−1^) ν_max_: 3260.17 (NH_2_), 1640.84 (C=O), 1579.82 (CH=N), 1285.36 (C-O). ^1^H-NMR δ: 11.11 (s, 1H, NH), 9.03 (s, 1H, -CH=), 8.14–8.16 (dd, *J* = 1.5 and 7.9 Hz, 1H, H-5, chromone ring), 8.10 (s, 1H, H-2, chromone ring), 7.85–7.88 (m, 1H, H-7, chromone ring), 7.73–7.75 (d, *J* = 8.3 Hz, 1H, H-8, chromone ring), 7.59 (s, 1H, H-6, chromone ring), 7.55–7.56 (m, 2H, H-2, H-6, phenyl), 7.35 (s, 1H, H-4, phenyl). ^13^C-NMR δ: 198.7 (-CF_3_), 192.6 (-CF_3_), 175.0 (C=O), 166.0 (Ar-Cq), 154.5 (CH), 138.8, 135.0 (=CH-), 131.8 (CH), 126.4 (Cq), 125.7 (CH), 123.8 (CH), 119.5 (Cq), 119.2 (CH), 112.1 (CH), 104.8, (Ar-CH), 100.9 (Ar-CH). HRMS (ESI) *m*/*z* calcd. for C_18_H_11_F_6_N_2_O_2_ (M + H)^+^: 401.0725, found 401.0730.

*4-Fluorobenzoic acid [1-(4-oxo-4H-chromen-3-yl)-meth-(E)-ylidene]-hydrazide* (**5**). Yellow solid. Yield: 65%. m.p. 201–202 °C. Anal. calcd. for C_17_H_11_FN_2_O_3_: C, 65.81; H, 3.57; N, 9.03. Found: C, 65.45; H, 3.51; N, 8.87%. FT IR (KBr, cm^−1^) ν_max_: 1642.11 (C=O), 1562.88 (CH=N), 1290.94 (C-O). ^1^H-NMR δ: 11.96 (s, 1H, NH), 8.85 (s, 1H, -CH=), 8.65 (s, 1H, H-2, chromone ring), 8.15–8.16 (dd, *J* = 1.1 and 7.9 Hz, 1H, H-5, chromone ring), 8.02–8.04 (dd, *J* = 5.6 and 8.3 Hz, 2H, H-2, H-6, phenyl), 7.87–7.90 (m, 1H, H-7, chromone ring), 7.74–7.76 (d, *J* = 8.2 Hz, 1H, H-8, chromone ring), 7.56–7.59 (t, 1H, H-6, chromone ring), 7.37–7.34 (t, 2H, H-3, H-5, phenyl). ^13^C-NMR δ: 175.5 (C=O), 165.5 (Ar-CF), 163.9 (C=O), 162.4 (Cq), 156.3 (CH), 155.0 (Ar-CH), 141.0 (Ar-CH), 135.2 (=CH-), 130.9 (CH), 130.1 (CH), 126.6 (Cq), 125.7 (CH), 123.8 (CH), 119.2 (Cq), 118.8 (CH), 116.0 (Ar-CH), 115.9 (Ar-CH). HRMS (ESI) *m*/*z* calcd. for C_17_H_12_FN_2_O_3_ (M + H)^+^: 311.0840, found 311.0832.

*3-[(2,3,5,6-Tetrafluorophenyl)-hydrazonomethyl]-chromen-4-one* (**6**). White solid. Yield: 72%. m.p. 241–242 °C. Anal. calcd. for C_16_H_8_F_4_N_2_O_2_: C, 57.15; H, 2.40; N, 8.33. Found: C, 56.94 H, 2.12; N, 8.07%. FT IR (KBr, cm^−1^) ν_max_: 3243.32 (NH_2_), 1641.28 (C=O), 1517.95 (CH=N), 1226.21 (C-O). ^1^H-NMR δ: 10.58 (s, 1H, NH), 8.63 (s, 1H, -CH=), 8.31 (s, 1H, H-2, chromone ring), 8.14–8.15 (dd, *J* = 1.5 and 7.9 Hz, 1H, H-5, chromone ring), 7.85–7.88 (m, 1H, H-7, chromone ring), 7.71–7.73 (d, *J* = 7.9 Hz, 1H, H-8, chromone ring), 7.54–7.57 (m, 1H, H-6, chromone ring), 7.24–7.29 (m, 1H, H-4, phenyl). ^13^C-NMR δ: 175.4 (C=O), 156.3 (CH), 153.4 (Ar-CF), 135.0 (Ar-CF), 134.8 (=CH-), 126.4 (Cq), 125.7 (CH), 123.8 (CH), 119.4 (Cq), 119.1 (CH), 96.3 (Ar-CH). HRMS (ESI) *m*/*z* calcd. for C_16_H_9_F_4_N_2_O_2_ (M + H)^+^: 337.0600, found 337.0604.

*3-[(5-Fluoro-2-methylphenyl)-hydrazonomethyl]-chromen-4-one* (**7**). White solid. Yield: 80%. m.p. 224–225 °C. Anal. calcd. for C_17_H_13_FN_2_O_2_: C, 68.91; H, 4.42; N, 9.45. Found: C, 69.01; H, 4.19; N, 9.12%. IR (KBr, cm^−1^) ν_max_: 3312.09 (NH_2_), 1639.40 (C=O), 1537.14 (CH=N), 1281.08 (C-O). ^1^H-NMR δ: 9.88 (s, 1H, NH), 8.93 (s, 1H, -CH=), 8.34 (s, 1H, H-2, chromone ring), 8.15–8.16 (dd, *J* = 1.5 and 7.9 Hz, 1H, H-5, chromone ring), 7.84–7.86 (m, 1H, H-7, chromone ring), 7.72–7.74 (d, *J* = 8.3 Hz, 1H, H-8, chromone ring), 7.54–7.56 (t, 1H, H-6, chromone ring), 7.23–7.25 (dd, *J* = 2.6 and 11.7 Hz, 1H, H-6, phenyl), 7.04–7.07 (t, 1H, H-3, phenyl), 6.47–6.50 (m, 1H, H-4, phenyl), 2.19 (s, 3H, CH_3_). ^13^C-NMR δ: 175.5 (C=O), 161.6 (Ar-CF), 156.3 (Cq), 153.5 (Ar-Cq), 145.0 (Ar-Cq), 134.8 (=CH-), 131.8 (CH), 131.6 (CH), 126.2 (Cq), 125.7 (CH), 123.8 (CH), 119.9 (Cq), 119.1 (CH), 116.9 (Ar-CH), 105.1 (Ar-CH), 95.4 (Ar-CH), 17.3 (-CH_3_). HRMS (ESI) *m*/*z* calcd. for C_17_H_14_FN_2_O_2_ (M + H)^+^: 297.1047, found 297.1039.

*3-[(2,4,6-Trifluorophenyl)-hydrazonomethyl]-chromen-4-one* (**8**). Dark yellow solid. Yield: 64%. m.p. 211–212 °C. Anal. calcd. for C_16_H_9_F_3_N_2_O_2_: C, 60.38; H, 2.85; N, 8.80. Found: C, 60.65; H, 2.52; N, 8.59%. FT IR (KBr, cm^−1^) ν_max_: 3247.92 (NH_2_), 1644.23 (C=O), 1536.56 (CH=N), 1223.66 (C-O). ^1^H-NMR δ: 9.77 (s, 1H, NH), 8.58 (s, 1H, -CH=), 8.12–8.14 (dd, *J* = 1.5 and 7.9 Hz, 1H, H-5, chromone ring), 8.10 (s, 1H, H-2, chromone ring), 7.83–7.86 (m, 1H, H-7, chromone ring), 7.69–7.70 (d, *J* = 8.3 Hz, 1H, H-8, chromone ring), 7.53–7.55 (m, 1H, H-6, chromone ring), 7.19–7.24 (m, 2H, H-3, H-5, phenyl). ^13^C-NMR δ: 175.4 (C=O), 164.0 (Ar-CF), 156.1 (Ar-CF), 135.7 (=CH-), 127.2 (Cq), 125.8 (Cq), 125.2 (CH), 120.5 (Cq), 119.4 (CH). HRMS (ESI) *m*/*z* calcd. for C_16_H_10_F_3_N_2_O_2_ (M + H)^+^: 319.0694, found 319.0707.

*3-(Pentafluorophenyl-hydrazonomethyl)-chromen-4-one* (**9**). Yellow solid. Yield: 70%. m.p. 245–246 °C. Anal. calcd. for C_16_H_7_F_5_N_2_O_2_: C, 54.25; H, 1.99; N, 7.91. Found: C, 54.43; H, 2.05; N, 7.85%. FT IR (KBr, cm^−1^) ν_max_: 3271.30 (NH_2_), 1620.34 (C=O), 1524.56 (CH=N), 1272.51 (C-O). ^1^H-NMR δ: 10.41 (s, 1H, NH), 8.61 (s, 1H, -CH=), 8.28 (s, 1H, H-2, chromone ring), 8.13–8.15 (dd, *J* = 1.9 and 8.1 Hz, 1H, H-5, chromone ring), 7.84–7.87 (m, 1H, H-7, chromone ring), 7.70–7.72 (d, *J* = 8.7 Hz, 1H, H-8, chromone ring), 7.54–7.56 (m, 1H, H-6, chromone ring). ^13^C-NMR δ: 175.4 (C=O), 156.3 (CH), 153.3 (Ar-CH), 135.0 (=CH-), 134.8 (CH), 126.4 (Cq), 125.7 (CH), 123.8 (CH), 119.3 (Cq), 119.2 (CH). HRMS (ESI) *m*/*z* calcd. for C_16_H_8_F_5_N_2_O_2_ (M + H)^+^: 355.0506, found 355.0513.

*3-[(2-Trifluoromethylphenyl)-hydrazonomethyl]-chromen-4-one* (**10**). Yellow solid. Yield: 75%. m.p. 233–235 °C. Anal. calcd. for C_17_H_11_F_3_N_2_O_2_: C, 61.45; H, 3.34, N, 8.43. Found: C 61.23; H, 3.15; N, 8.55%. FT IR (KBr, cm^−1^) ν_max_: 3319.96 (NH_2_), 1641.56 (C=O), 1584.02 (CH=N), 1274.36 (C-O). ^1^H-NMR δ: 9.98 (s, 1H, NH), 8.88 (s, 1H, -CH=), 8.51 (s, 1H, H-2, chromone ring), 8.15–8.17 (dd, *J* = 1.5 and 7.9 Hz, 1H, H-5, chromone ring), 7.84–7.86 (m, 1H, H-7, chromone ring), 7.83–7.82 (d, *J* = 8.2 Hz, 1H, H-8, chromone ring), 7.72–7.74 (m, 1H, H-6, chromone ring), 7.53–7.57 (m, 3H, H-3, H-5, H-6, phenyl), 6.95–6.96 (m, 1H, H-4, phenyl). ^13^C-NMR δ: 188.8 (-CF_3_), 175.5 (C=O), 159.6 (Ar-Cq), 156.2 (CH), 153.8 (Ar-Cq), 142.8 (CH), 137.8 (Ar-CH), 134.9 (=CH-), 133.8 (CH), 126.3 (Cq), 125.7 (CH), 124.0 (CH), 122.5 (Cq), 119.3 (Cq), 119.1 (CH), 115.7 (Ar-CH), 111.9 (Ar-CH). HRMS (ESI) *m*/*z* calcd. for C_17_H_12_F_3_N_2_O_2_ (M + H)^+^: 333.0851, found 333.0858.

*Complex of 4-Fluorobenzoic acid [1-(4-oxo-4H-chromen-3-yl)-meth-(E)-ylidene]-hydrazide with Cu (II)* (**11**). Green solid. Yield: 65%. m.p. > 300 °C. Anal. calcd. for C_17_H_11_ClCuFN_2_O_3_: C, 49.88; H, 2.69; N, 6.85. Found: C, 50.07; H, 3.07, N: 6.32%. FT IR (KBr, cm^−^^1^) ν_max_: 1636.91 (C=O), 1573.46 (CH=N), 1239.25 (C-O), 614.26 (Cu-N). HRMS (ESI) *m*/*z* calcd. for C_17_H_12_ClCuFN_2_O_3_ (M + H)^+^: 410.9973, found 411.0966.

### 3.5. X-ray Diffraction Studies

Single crystal X-ray data were collected on a micro-focus SuperNova diffractometer (Rigaku Oxford Diffraction, Yarnton, UK) with an Atlas detector using MoK_α_ and ω scan. The measurements were carried out at low temperature for **1**, **8** and **10** whereas at room temperature for **3** and **5**. The X-ray data were corrected for absorption [36]. All structures were solved using SHELXT [37] and refined with SHELXL-2014/7 [38]. The crystallographic programs PLATON [39] and MERCURY [40] were used for structure analysis and presentation of the results. Crystallographic data and the final figures of merit for structure refinements are summarized in Table 1. Crystallographic data for the structural analyses of **1**, **3**, **5**, **8** and **10** have been deposited with the Cambridge Crystallographic Data Centre (CCDC nos.1851685–1851689). These data can be obtained free of charge via http://www.ccdc.cam.ac.uk/conts/retrieving.html (or from the CCDC, 12 Union Road, Cambridge CB2 1EZ, UK; Fax: +44-1223-336-033; E-mail: deposit@ccdc.cam.ac.uk).

### 3.6. Research on Antimicrobial Potential (Initial Determination)

The microbial reference strains representing different families of bacteria and fungi, pathogenic for man and animals, were used. The 15 strains were derived from international microbial collections held by the Department of Pharmaceutical Microbiology and Microbiological Diagnostics of the Medical University of Lodz: *Candida albicans* ATCC 10231, *Enterococcus faecalis* ATCC 29212, *Klebsiella pneumoniae* ATCC 700603, *Pseudomonas aeruginosa* NCTC 6749, *Proteus vulgaris* CCM 1799, *Staphylococcus epidermidis* ATCC 12228, *Shigella flexneri* ATCC 12022, *Streptococcus pneumoniae* ATCC 49619, *Streptococcus pyogenes* ATCC 19615, *Corynebacterium jeikeium* K411, *Escherichia coli* ATCC 25922, *Staphylococcus aureus* NCTC 4135, *Streptococcus dysgalactiae subsp. equisimilis* ATCC 12394, *Neisseria meningitidis* DSM 25942, *Listeria monocytogenes* PCM 2191.

The strains were stored at −80 °C, frozen in a 1:1 mixture of Brain Heart Infusion (BHI) broth and glycerol. The reference strains, depending on the growth requirements, were grown on a solid medium—nutrient agar, nutrient agar with 5% addition of lambs defibrinated blood or (in the case of *Corynebacterium jeikeium*) Triptic Soy Agar (TSA) with the addition of sheep blood, yeast extract and Tween-80. The susceptibility of the strains to chemical compounds was measured analogously to the adopted method of diffusion and disc preparation of antibiotics for compounds introduced into treatment [41]. After receiving the pure culture of the reference strain, obtained from frozen samples and grown on the appropriate medium for growth requirements, suspensions of the tested strains were prepared in a physiological NaCl solution with a density of 0.5 on the McFarland scale. These suspensions were seeded with a cotton ball over the entire surface of the test medium. Discs impregnated with the test compound at a concentration of 100 or 200 µg/mL were then placed on it and incubated for 24 h at 37 °C.

### 3.7. XTT-Assay—Cell Culture

The mouse fibroblast cell line L929 (Sigma-Aldrich) was grown in MEM (Minimum Essential Medium) (Biowest, Nuaillé, France) containing 10% of Fetal Bovine Serum (Biowest), 1 mmol/L sodium pyruvate (Biological Industries, Cromwell, CT, USA), 0.5 mmol/L non-essential amino acids (Sigma-Aldrich) and 100 units/mL penicillin with 100 mg/mL streptomycin (Biological Industries) in an incubator at 37 °C with 5% CO_2_. Cells were plated and grown to 80% confluence before the initiation of the assay.

The EA.hy926 cell line (the human umbilical vein, somatic cell hybrid, American Type Culture Collection, Manassas, VA, USA) was used to evaluate the cytotoxicity of novel compounds. Cells were grown in Dulbecco’s Modified Eagle’s Medium (DMEM) which contains 10% of Fetal Bovine Serum (Biowest), 2 mmol/L glutamine (Sigma-Aldrich) and 100 units/mL penicillin with 100 mg/mL streptomycin (Biological Industries). Cells were plated and grown in an incubator at 37 °C with 5% CO_2_ to 80% confluence before the initiation of the assay.

### 3.8. XTT Cytotoxicity Assay

The XTT assay was used to evaluate cell metabolic activity [42,43]. In viable cells, 2,3-bis-(2-methoxy-4-nitro-5-sulphophenyl)-2H-tetrazolium-5-carboxanilide (XTT) is metabolically reduced to a water-soluble orange formazan product. The number of viable cells is correlated with the colour intensity determined by photometric measurements. XTT (Serva, Heidelberg, Germany) was dissolved in MEM without phenol red (PAN-Biotech, Aidenbach, Germany) or DMEM without phenol red (PAN-Biotech) at a concentration of 1 mg/mL. Phenazine metosulphate (PMS) (Serva) was dissolved in phosphate-buffered saline (PBS, 5 mmol/L). PMS solution was then added to the XTT solution at a concentration of 25 μM. Investigated compounds were dissolved in dimethyl sulfoxide (DMSO) to obtain stock solutions.

L929 and EA.hy926 cells were seeded into 96-well plates at a density of 1 × 10^4^ cells per well. They were cultured for 24 h in the incubator (37 °C and 5% CO_2_). After that time, the medium was removed. Then the 100 µL of medium containing investigated compounds (in the range of concentration 0.01–1250 µmol/L) or only culture medium (blank control) were added. Cisplatin was used as a reference compound. Cells were further incubated for 24 h. At the third day, the old medium were removed and cells were washed with PBS. 100 µL of culture medium with the additional 50 µL of the XTT/PMS solution were added to each well. Plates were incubated in the dark for 3 h at 37 °C. Then, the plates were swayed and an aliquot of 100 µL from each well was transferred into the corresponding well of a new plate. Finally, the absorbance was measured by using the microplate reader (Synergy H1, BioTek, Winooski, VT, USA) at a wavelength of 450 nm. The cell viability was expressed as a percentage of the control values (blanks).

### 3.9. Statistical Analysis of Cell Proliferation

All statistical analyses were performed using Statistica (version 13.1.336.0 PL, DELL Inc., Tulsa, OK, USA) software [44]. The data was analysed using the *t*-test and the Shapiro-Wilk test. The significance levels were set at *p* < 0.05 for the t-test and *p* > 0.05 for the Shapiro-Wilk test.

## 4. Conclusions

The presented method for synthesizing hydrazine or hydrazide derivatives of 3-formylchromone with fluorine substituents resulted in the creation of novel ligands which can be used to synthesize the respective complexes with metal ions. Eight hydrazine and two hydrazide derivatives of chromone with fluorine substituents were obtained as a result of chemical condensation of 3-formylchromone with respective hydrazines or hydrazide. X-ray single-crystal studies confirmed that in all structures, the hydrazine moiety is oriented similarly with respect to the chromone ring. The bond lengths and angles were found to be in good agreement to each other. The crystal packing appeared to be dominated by N-H···O interactions, with hydrogen-bonded dimers being the preferred structural motif. The reaction of the ligand with copper (II) chloride resulted in neutral mononuclear complex. All compounds described in this paper were initially tested for antimicrobial properties, and **1**, **4**, **7**, **9**, **10**, **11** were found to exhibit antimicrobial potential. Tested compounds were able to inhibit specific microbial growth during the experiment. A good number of synthesized compounds exhibited comparable or higher antiproliferative activities than the control drug cisplatin. **5** and **11** were found to possess IC_50_ values below 1 µmol/L on EA.hy926 cell line. This supports the hypothesis that hydrazide derivatives of 3-formylchromone constitute a class of potential antiproliferative agents.

## Figures and Tables

**Table 1:** Crystallographic data for **1**, **3**, **5**, **8** and **10**.

	1	3	5	8	10
Empirical formula	C_16_H_10_F_2_N_2_O_2_	C_17_H_11_F_3_N_2_O_2_	C_34_H_22_F_2_N_4_O_6_ H_2_O	C_16_H_9_F_3_N_2_O_2_	C_34_H_22_F_6_N_4_O_4_
Formula weight	300.26	332.28	638.57	318.25	664.55
Crystal system	Triclinic	Monoclinic	Monoclinic	Triclinic	monoclinic
Space group	*P*ī	*P*2_1_/c	*P*2_1_/n	*P*ī	*C*2/c
*a* (Å)	6.8791 (2)	16.8965 (9)	11.1731 (10)	3.9211 (1)	25.5373 (11)
*b* (Å)	12.0848 (4)	4.8728 (2)	16.4967 (12)	11.7020 (2)	7.4105 (3)
*c* (Å)	16.1485 (6)	18.4032 (10)	16.4583 (12)	14.2192 (3)	14.7383 (5)
α (°)	94.432 (3)	90	90	88.791 (2)	90
β (°)	100.817 (3)	105.343 (5)	109.125 (9)	87.938 (2)	93.014 (3)
γ (°)	103.431 (3)	90	90	87.939(2)	90
*V* (Å^3^)	1272.24 (8)	1461.19 (13)	2866.1 (4)	651.47 (2)	2785.28 (19)
*Z*	4	4	4	2	4
*T* (K)	100 (1)	290 (1)	293 (1)	100 (1)	91 (1)
*F* (000)	616	680	1320	324	1360
*D_x_* (g cm^−3^)	1.568	1.510	1.480	1.622	1.585
*µ* (mm^−1^)	0.125	0.126	0.114	0.138	0.132
Wavelength (Å)	0.71073	0.71073	0.71073	0.71073	0.71073
*θ* range (°)	3.11–30.0	3.75–30.0	3.13–30.0	3.32–30.0	3.12–30.0
Measured reflections	14, 205	18, 926	30, 676	8452	14, 914
Unique reflections	7388	4247	8335	3806	4067
Observed reflections [*I* > 2*σ* (*I*)]	5176	2004	3518	3359	2917
Completeness to *θ*_max_ (%)	99.5	99.8	99.8	99.7	99.8
Parameters/restraints	405/0	249/93	441/0	212/0	244/0
*R* [*I* > 2*σ* (*I*)]	0.0481	0.0696	0.0607	0.0336	0.0518
*wR* (all data)	0.1372	0.2536	0.1905	0.1016	0.1434
*S*	1.006	1.030	1.011	1.062	1.058
Δ*ρ*_max_ (e Å^−3^)	0.494	0.299	0.216	0.443	0.561
Δ*ρ*_min_ (e Å^−3^)	−0.278	−0.365	−0.214	−0.194	−0.490

**Table 2:** Hydrogen-bonding geometries (Å,°) for **1**, **3**, **5**, **8** and **10**.

Comp.	H–Bond	D–H	H⋅⋅⋅A	D⋅⋅⋅A	D–H⋅⋅⋅A
**1**	N2–H2A⋅⋅⋅F4	0.90 (2)	2.45 (2)	2.937 (2)	114 (2)
	N2–H2A⋅⋅⋅O4^i^	0.90 (2)	2.28 (2)	3.091 (2)	150 (2)
	N4–H4A⋅⋅⋅O2^i^	0.86 (2)	2.15 (2)	2.957 (2)	158 (2)
**3**	N2–H2A⋅⋅⋅O2^i^	0.91 (3)	2.00 (3)	2.903 (3)	172 (2)
**5**	N2–H2A⋅⋅⋅O6^i^	0.83 (2)	2.11 (2)	2.910 (2)	163 (2)
	N4–H4A⋅⋅⋅O7	0.90 (2)	1.91 (3)	2.785 (2)	162 (2)
	O7–H7A⋅⋅⋅O3	0.95 (3)	1.83 (3)	2.750 (2)	162 (2)
	O7−H7A⋅⋅⋅N1	0.95 (3)	2.50 (3)	3.143 (2)	125 (2)
	O7–H7B⋅⋅⋅O6^ii^	0.87 (4)	2.17 (4)	2.929 (3)	145 (3)
	O7–H7B⋅⋅⋅N3^ii^	0.87 (4)	2.42 (4)	3.061 (3)	131 (3)
**8**	N2–H2A⋅⋅⋅O2^i^	0.85 (2)	2.10 (2)	2.926 (1)	163 (2)
**10**	N2–H2A⋅⋅⋅O2^i^	0.90 (2)	2.32 (2)	3.150 (2)	154 (2)
	N2–H2A⋅⋅⋅F1	0.90 (2)	2.27 (2)	2.849 (2)	122 (2)

Symmetry codes: **1** (i) 1−*x*, 2−*y*, 1−*z*; **3** (i) −*x*+1, −*y*+2, −*z*+1; **5** (i) −1+*x*, *y*, *z*; (ii) 1−*x*, −*y*, 1−*z;* **8** (i) 1−*x*, 1−*y*, 1−*z;* **10** (i) 1−*x*, *y*, ½−*z*.

**Table 3:** Inhibition of growth zones (lowest effective studied concentration).

Strain	1	4	7	9	10	11
*S. epidermidis*		100 µg/mL	100 µg/mL		100 µg/mL	100 µg/mL
*S. pneumoniae*	100 µg/mL					
*S. pyogenes*						200 µg/mL
*S. aureus*						100 µg/mL
*N. meningitidis*				100 µg/mL *		

* *N. meningitidis*, the action of 9 was noted just for 100 µg/mL and the influence in concentration of 200 µg/mL was not observed.

**Table 4:** Cell proliferation study results.

Compound	IC_50_ [µmol/L]		The Range of Influence [%, Control = 100%]	
	L929	EA.hy926	L929	EA.hy926
Cisplatin	74.44	67.36	8.49–55.70	8.71–75.14
**1**	208.51	59.15	27.64–101.8	14.79–106.26
**3**	3714.50	975.09	55.91–96.98	37.40–101.33
**4**	1034.69	533.79	45.61–101.31	28.46–102.18
**5**	65.92	0.18	16.03–56.35	11.81–60.64
**6**	727.78	578.25	40.05–93.11	24.47–97.86
**7**	953.76	953.37	44.89–96.11	42.43–101.05
**8**	98.49	18.12	38.11–64.40	23.31–63.94
**9**	385.65	253.00	13.92–85.11	13.15–69.88
**10**	1207.48	645.48	48.62–73.47	31.21–94.69
**11**	35.01	0.04	10.30–75.02	8.58–53.23

## Supplementary Materials

The following are available online. Table S1: Selected geometric parameters (Å,°) for **1**, **3**, **5**, **8** and **10**. Table S2: Aromatic π⋯π interactions (Å,°) for structures **1**, **5**, **8**, **10**. Figure S3: Cell proliferation study concentration-response curves for tested compounds on cell lines: L929 EA.hy926. Figure S4: ^1^H-NMR and ^13^C-NMR Spectra.
