# Supplementary material for: Synthesis, Spectroscopic Analysis and Assessment of the Biological Activity of New Hydrazine and Hydrazide Derivatives of 3-Formylchromone
Source: Molecules. 2018 Aug 17;23(8):2067. doi: 10.3390/molecules23082067 (PMC6222780; doi:10.3390/molecules23082067)

molecules

Supplementary Materials

Article

Synthesis, spectroscopic analysis and assessment of the biological activity of new hydrazine and hydrazide derivatives of 3-formylchromone

Krzysztof Słomiak [1,*], Andrzej Łazarenkow [1], Lilianna Chęcińska [2], Joachim Kusz [3], Justyn Ochocki [1] and Jolanta Nawrot-Modranka [1]

1 Department of Bioinorganic Chemistry, Medical University of Lodz, Muszyńskiego 1, 90-151 Łódź, Poland; andrzej.lazarenkow@umed.lodz.pl (A.Ł); justyn.ochocki@umed.lodz.pl (J.O.); jolanta.nawrot-modranka@umed.lodz.pl (J.N-M.)
2 Department of Physical Chemistry, Faculty of Chemistry, University of Lodz, Pomorska 163/165, 90-236 Łódź, Poland; lilianna.checinska@chemia.uni.lodz.pl
3 Department of Physics of Crystals, Institute of Physics, University of Silesia, Uniwersytecka 4, 40-007 Katowice, Poland; joachim.kusz@us.edu.pl
* Correspondence: krzysztof.slomiak@umed.lodz.pl; Tel.: +48-42-677-92-20

Received: date; Accepted: date; Published: date

Table of contents

Table S1. Selected geometric parameters for **1**, **3**, **5**, **8** and **10.** page 2

Table S2: Aromatic π···π interactions (Å,°) for structures **1**, **5**, **8**, **10.** page 3

Figure S3: Cell proliferation study concentration-response curves for tested compounds on page 4

cell lines: L929 EA.hy926.

Figure S4: ^{1}H NMR and ^{13}C NMR Spectra page 12

Table S1. Selected geometric parameters (Å,°) for **1**, **3**, **5**, **8** and **10.**

	**1**	**1**	**3**	**5**	**5**	**8**	**10**
O1–C2	1.3476(16)	1.3477(17)	1.348(3)	1.341(3)	1.340(2)	1.3473(11)	1.313(2)#
O1–C9	1.3736(16)	1.3766(17)	1.359(3)	1.370(3)	1.374(3)	1.3738(10)	1.373(2)
O2–C4	1.2360(16)	1.2362(16)	1.234(3)	1.226(2)	1.229(2)	1.2364(10)	1.234(2)
C2–C3	1.3498(19)	1.3502(19)	1.336(3)	1.336(3)	1.333(3)	1.3550(12)	1.347(3)#
C3–C4	1.4544(18)	1.4594(19)	1.445(3)	1.449(3)	1.445(3)	1.4611(11)	1.433(3)#
C4–C10	1.4622(18)	1.4701(19)	1.454(3)	1.462(3)	1.458(3)	1.4651(12)	1.458(2)
C9–C10	1.3891(19)	1.3870(19)	1.380(3)	1.381(3)	1.377(3)	1.3944(11)	1.394(2)
C3–C11	1.4622(18)	1.4644(19)	1.444(3)	1.453(3)	1.451(3)	1.4628(12)	1.480(3)#
N1–N2	1.3608(16)	1.3677(16)	1.350(3)	1.369(2)	1.383(2)	1.3711(10)	1.364(2)
N1–C11	1.2831(18)	1.2811(18)	1.278(3)	1.268(3)	1.266(3)	1.2861(11)	1.279(2)
N2–C12*	1.3755(18)	1.3788(18)	1.372(3)	1.349(3)	1.339(3)	1.3905(11)	1.386(2)
O3–C12	–	–	–	1.223(2)	1.235(2)	–	–
C12–C13	–	–	–	1.472(3)	1.479(3)	–	–
C2–O1–C9	118.26(11)	118.38(11)	118.4(2)	118.3(2)	118.2(2)	118.45(7)	117.05(14) #
O1–C2–C3	125.24(13)	125.03(13)	125.4(2)	125.5(2)	125.3(2)	125.16(8)	126.62(9)#
C2–C3–C4	119.83(12)	119.85(13)	119.0(2)	119.7(2)	119.8(2)	119.45(8)	120.11(18)#
O2–C4–C3	123.04(12)	122.83(13)	121.9(2)	122.5(2)	122.4(2)	122.50(8)	122.77(17)#
C3–C4–C10	114.56(12)	114.67(12)	115.4(2)	114.6(2)	115.0(2)	114.99(7)	113.80(15)#
C2–C3–C11	121.07(12)	121.23(13)	121.1(2)	121.3(2)	121.8(2)	121.82(8)	120.60(18)
C3–C11–N1	119.49(13)	120.31(13)	120.9(2)	119.5(2)	121.6(2)	120.30(8)	121.20(15)
C11–N1–N2	116.58(12)	116.49(12)	117.4(2)	117.4(2)	114.6(2)	115.77(7)	116.56(14)
N1–N2–C12	118.37(12)	118.07(12)	120.3(2)	118.4(2)	119.5(2)	118.60(7)	117.71(14)
N2–C12–O3	–	–	–	121.9(2)	122.4(2)	–	–
C13–C12–O3	–	–	–	121.4(2)	121.8(2)	–	–
C2–C3–C11–N1	-0.2(2)	7.4(2)	4.7(4)	-19.6(3)	-25.4(3)	-3.86(13)	-11.0(3) #
C11–N1–N2–C12	-177.85(13)	172.82(13)	-178.5(2)	-177.0(2)	176.6(2)	-165.72(8)	-171.53(16)
N1–N2–C12–C13	164.16(13)	173.59(12)	-179.6(2)	-174.5(2)	-178.4(2)	-160.69(8)	-167.04(15)
O2–C4–C3–C11	0.5(2)	-0.1(2)	-0.1(4)	-3.3(3)	-2.5(3)	1.84(13)	179.35(17) #
N2–C12–C13–C14	-179.49(14)	-179.65(13)	-179.5(3)	25.4(3)	21.3(3)	179.77(8)	179.02(16)

* - for **5**, the N2–C12 bond differs in its type in comparison to the remaining structures;

# - for **10**, the geometric parameters involving the disordered C2 and C3 atoms are presented only for major component A.

Table S2: Aromatic π···π interactions (Å,°) for structures **1**, **5**, **8**, **10**.

Compound	Interaction	*Cg*(I)···*Cg*(J)	α	Cg(I)perp	*Cg*(J)perp	Slippage
**1**	*Cg*(1)···*Cg*(1)[i]	3.530(1)	0.0(1)	-3.281(1)	-3.381(1)	1.302
	Cg(1)···*Cg*(2)[i]	3.520(1)	2.3(1)	-3.269(1)	-3.314(1)	1.188
	Cg(3)···*Cg*(3)[ii]	3.805(1)	0.0(1)	-3.483(1)	-3.483(1)	1.531
	Cg(5)···*Cg*(7)[iii]	3.498(1)	5.5(1)	-3.422(1)	3.368(1)	0.947
	Cg(6)···*Cg*(7)[iv]	3.627(1)	3.8(1)	-3.327(1)	-3.402(1)	1.256
**5**	*Cg*(7)···*Cg*(5)[i]	3.726(1)	8.0(1)	-3.326(1)	-3.445(1)	1.419
**8**	*Cg*(2)···*Cg*(1)[i]	3.493(1)	1.1(1)	3.346(1)	-3.365(1)	0.937
	Cg(3)···*Cg*(3)[i]	3.921(1)	0.0(1)	3.552(1)	-3.552(1)	1.662
**10**	*Cg*(1)···*Cg*(1)[i]	3.415(1)	0.0(1)	3.409(1)	3.409(1)	0.187
	Cg(2)···*Cg*(3)[ii]	3.889(1)	3.3(1)	-3.523(1)	-3.533(1)	1.625

Cg···*Cg* – distance between ring centroids; α - dihedral angle between planes I and J; *Cg*(I)perp and *Cg*(J)perp - (interplanar spacing) perpendicular distance of *Cg*(I) on ring J and *Cg*(J) on ring I, respectively; slippage - distance between *Cg*(I) and perpendicular projection of *Cg*(J) on ring I.

Cg(1) (in molecule 1) and *Cg*(5) (in molecule 2) – a centre-of-gravity of heterocyclic ring; *Cg*(2) (in molecule 1) and *Cg*(6) (in molecule 2) – a centre-of-gravity of benzene ring (condensed with a heterocyclic one); *Cg*(3) (in molecule 1) and *Cg*(7) (in molecule 2) - a centre-of-gravity of phenyl ring.

Symmetry codes: **1** (i) -*x*,1-*y*,1-*z*; (ii) -*x*,1-*y*,-*z*; (iii) 1-*x*,2-*y*,1-*z*; (iv) -*x*,2-*y*,1-*z*; **5** (i) 1-*x*,-*y*,1-*z*; **8** (i) -1+*x*,*y*,*z*; **10** (i) 1-*x*,2-*y*,1-*z*; (ii) 1-*x*,1-*y*,1-*z*.

Figure S3: Cell proliferation study concentration-response curves for tested compounds on cell lines: L929 EA.hy926.

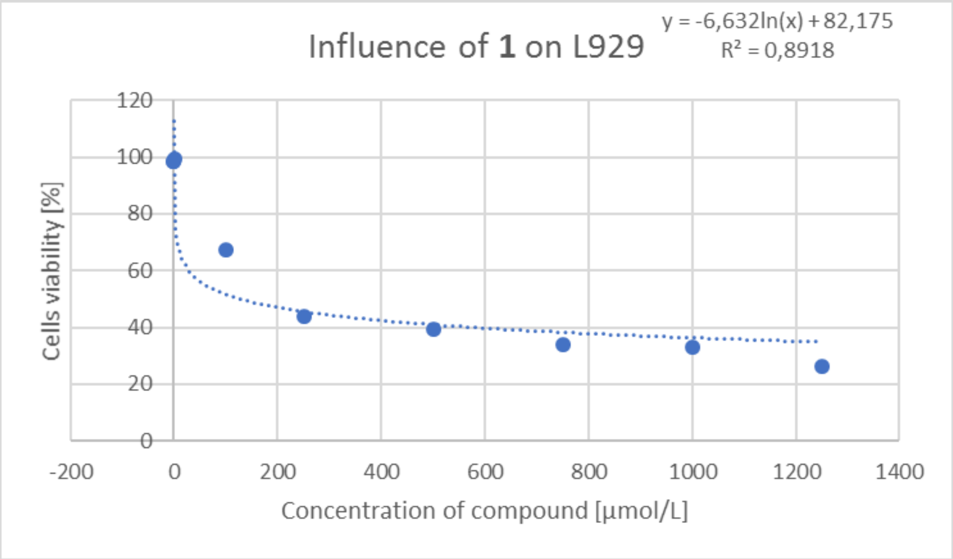

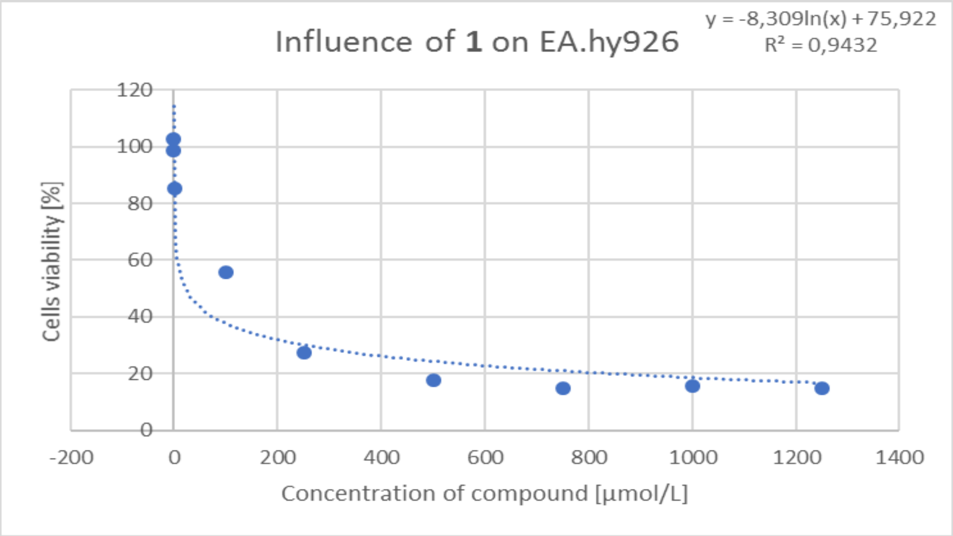

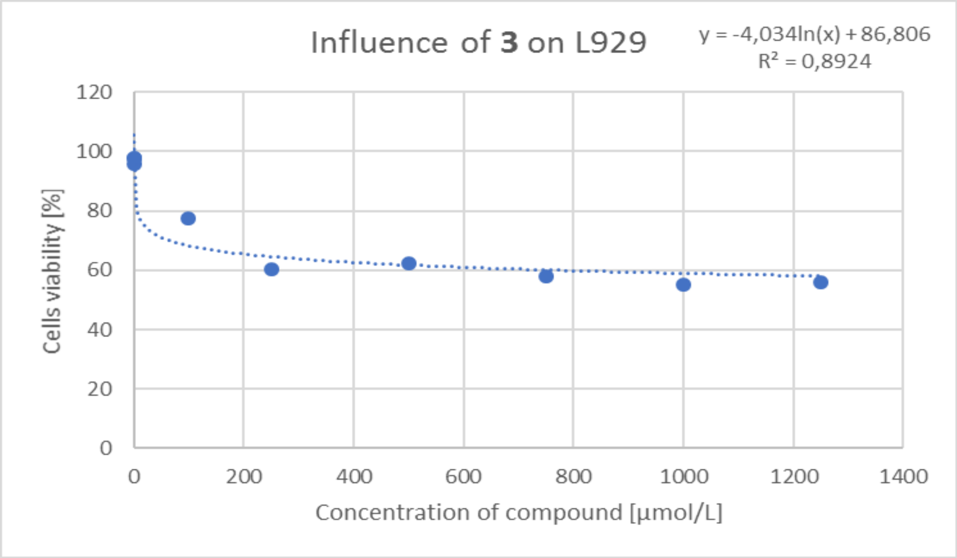

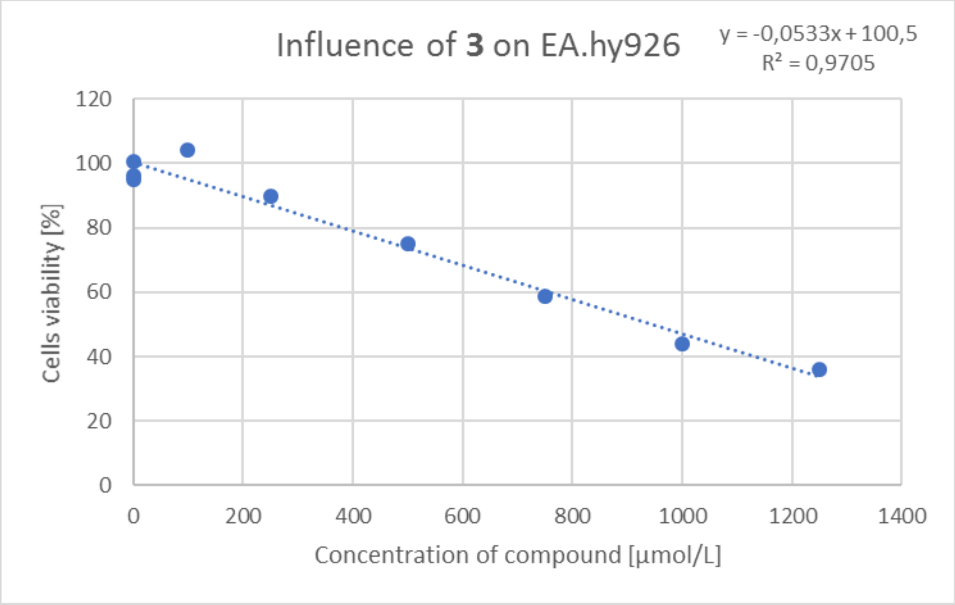
Influence of **3** on EA.hy926
y = -0,0533x + 100,5
R² = 0,9705
Cells viability [%]
120
100
80
60
40
20
0
0
200
400
600
800
1000
1200
1400
Concentration of compound [µmol/L]

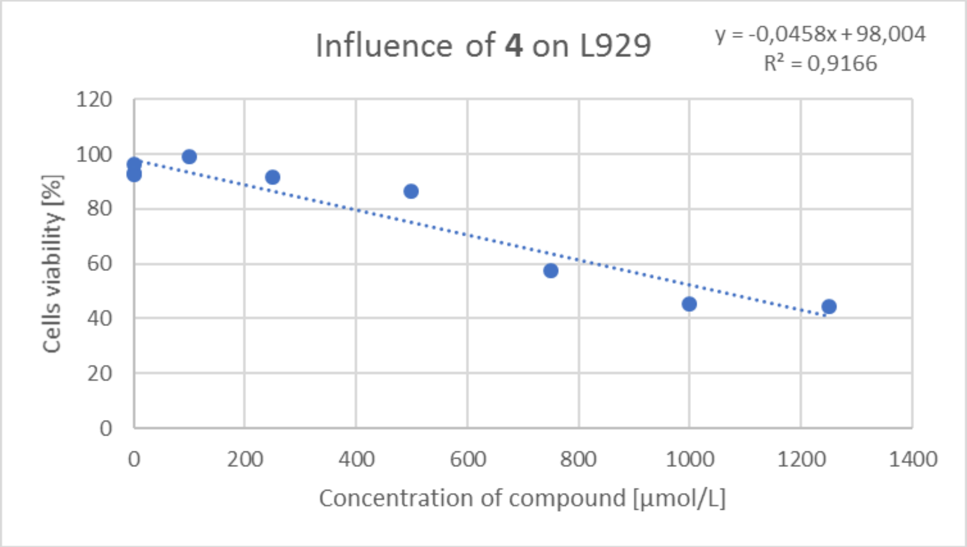
Influence of **4** on L929
y = -0,0458x + 98,004
R² = 0,9166
Cells viability [%]
120
100
80
60
40
20
0
0
200
400
600
800
1000
1200
1400
Concentration of compound [µmol/L]

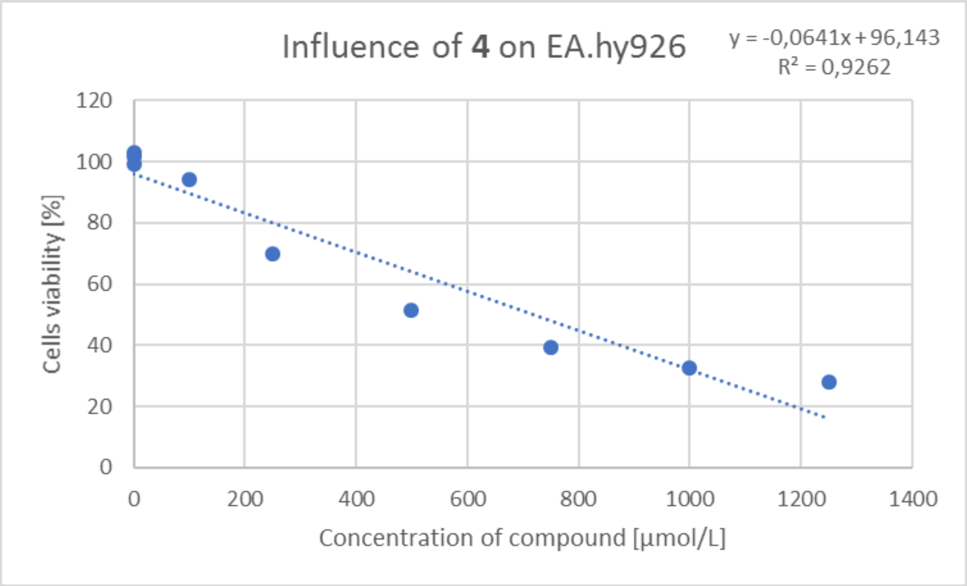
Influence of **4** on EA.hy926
y = -0,0641x + 96,143
R² = 0,9262
Cells viability [%]
120
100
80
60
40
20
0
0
200
400
600
800
1000
1200
1400
Concentration of compound [µmol/L]

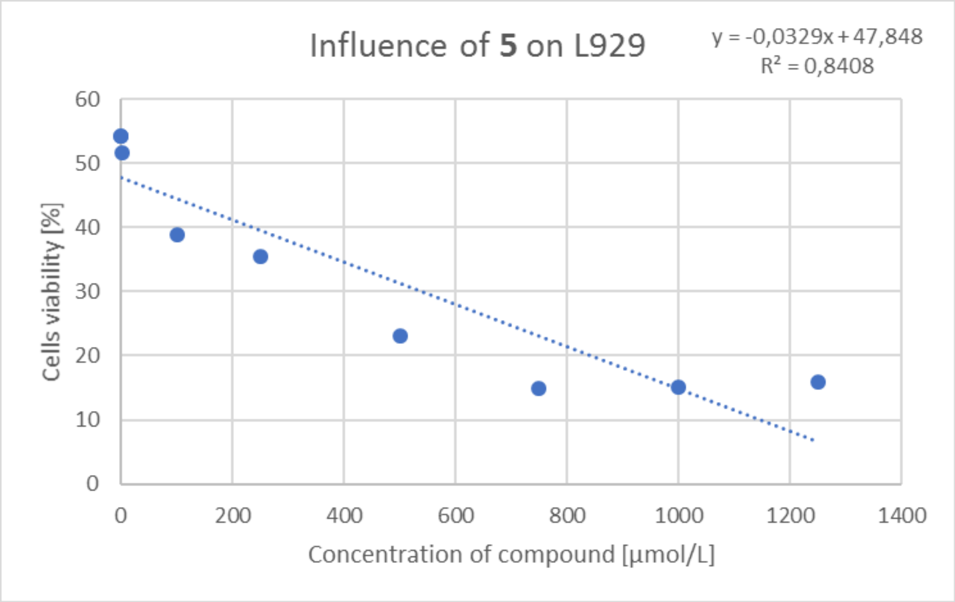
Influence of 5 on L929
y = -0,0329x + 47,848
R² = 0,8408
Cells viability [%]
60
50
40
30
20
10
0
0
200
400
600
800
1000
1200
1400
Concentration of compound [µmol/L]

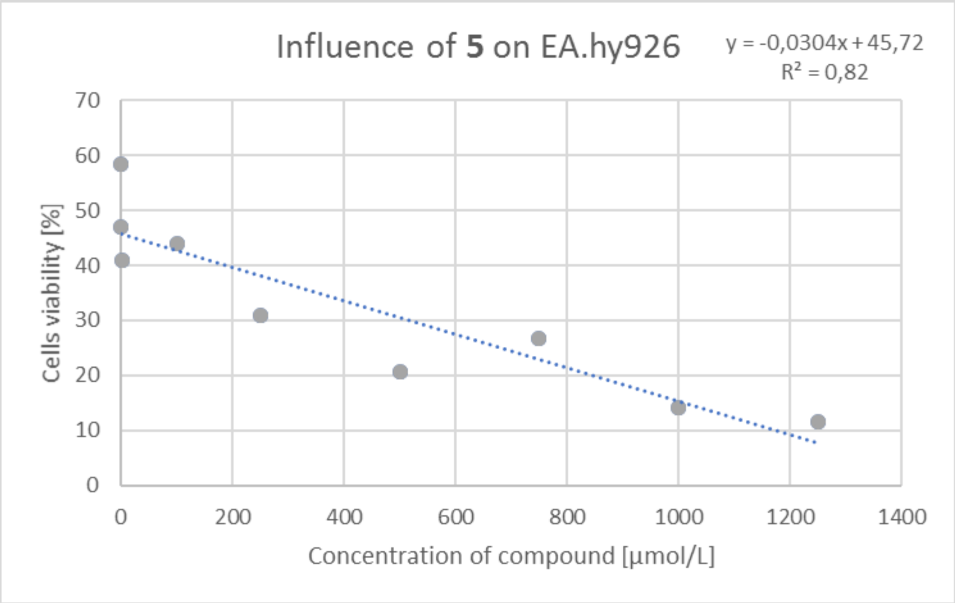
Influence of 5 on EA.hy926
y = -0,0304x + 45,72
R² = 0,82
Cells viability [%]
70
60
50
40
30
20
10
0
0
200
400
600
800
1000
1200
1400
Concentration of compound [µmol/L]

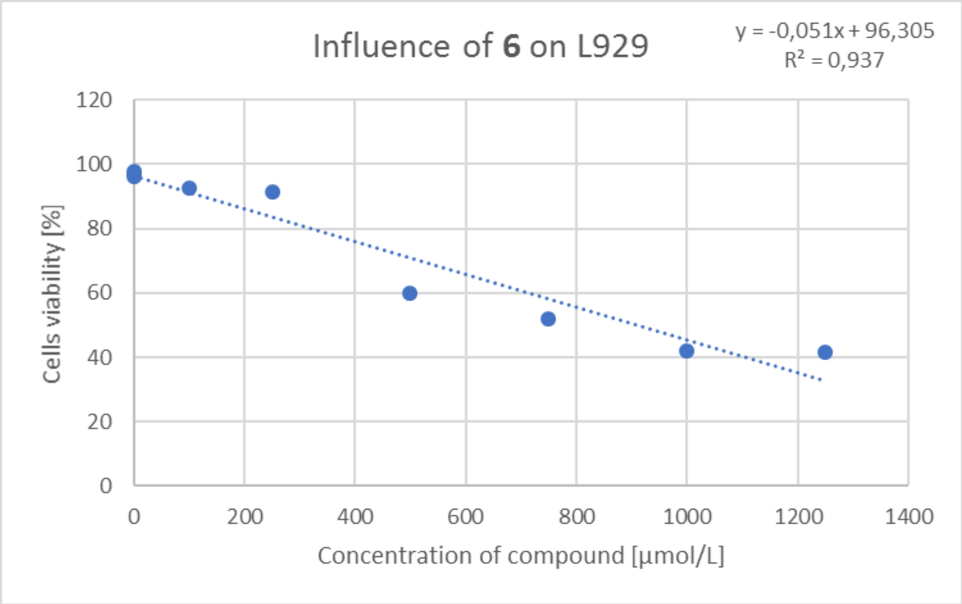
Influence of 6 on L929
y = -0,051x + 96,305
R² = 0,937
Cells viability [%]
120
100
80
60
40
20
0
0
200
400
600
800
1000
1200
1400
Concentration of compound [µmol/L]

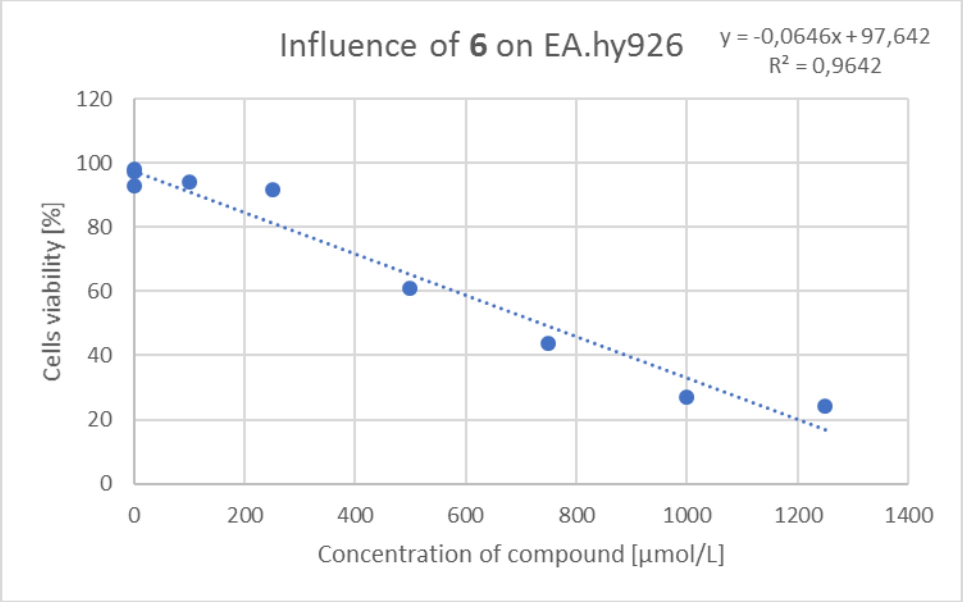
Influence of **6** on EA.hy926
y = -0,0646x + 97,642
R² = 0,9642
Cells viability [%]
Concentration of compound [µmol/L]
0
20
40
60
80
100
120
0
200
400
600
800
1000
1200
1400

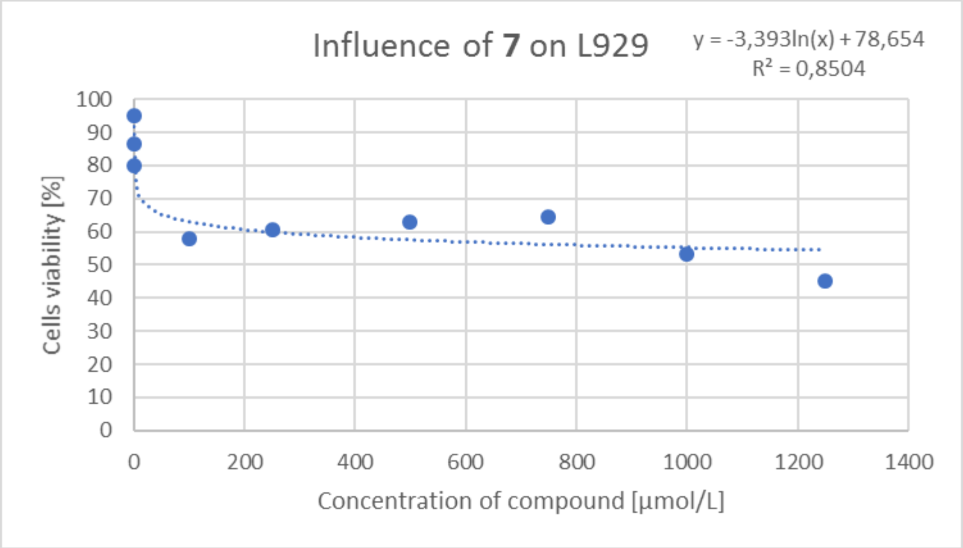
Influence of **7** on L929
y = -3,393ln(x) + 78,654
R² = 0,8504
Cells viability [%]
Concentration of compound [µmol/L]
0
10
20
30
40
50
60
70
80
90
100
0
200
400
600
800
1000
1200
1400

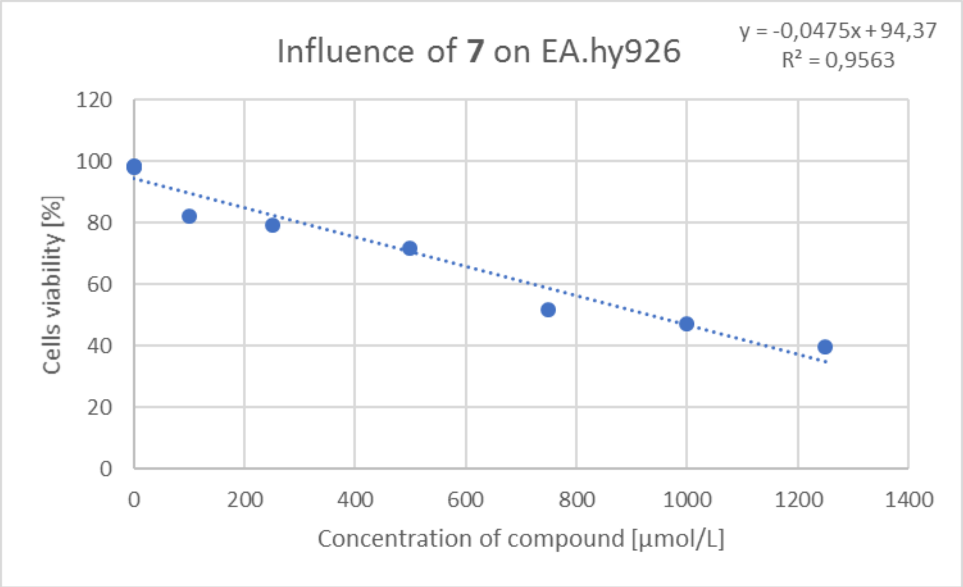
Influence of **7** on EA.hy926
y = -0,0475x + 94,37
R² = 0,9563
Cells viability [%]
Concentration of compound [µmol/L]
0
20
40
60
80
100
120
0
200
400
600
800
1000
1200
1400

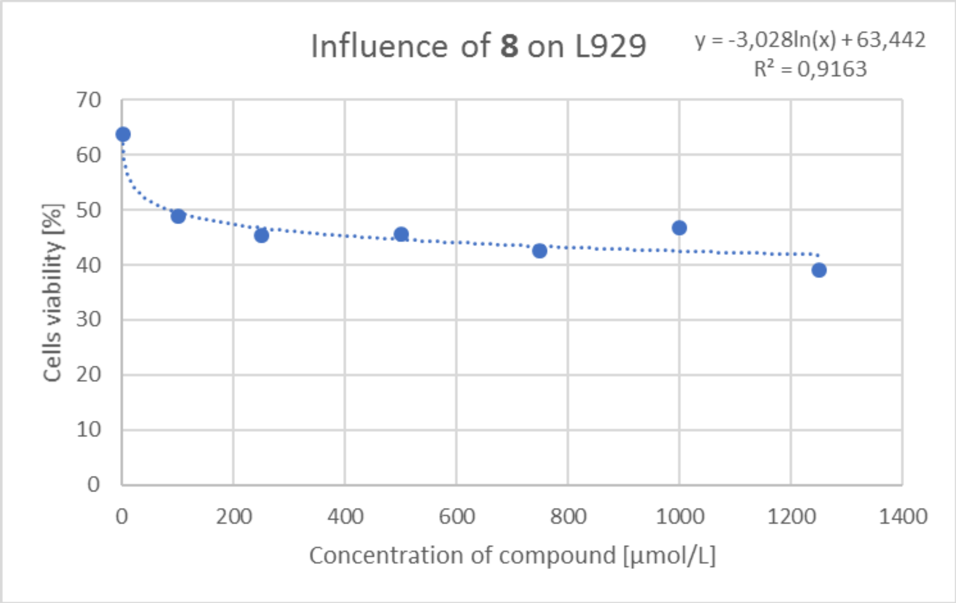
Influence of **8** on L929
y = -3,028ln(x) + 63,442
R² = 0,9163
Cells viability [%]
Concentration of compound [µmol/L]
0
10
20
30
40
50
60
70
0
200
400
600
800
1000
1200
1400

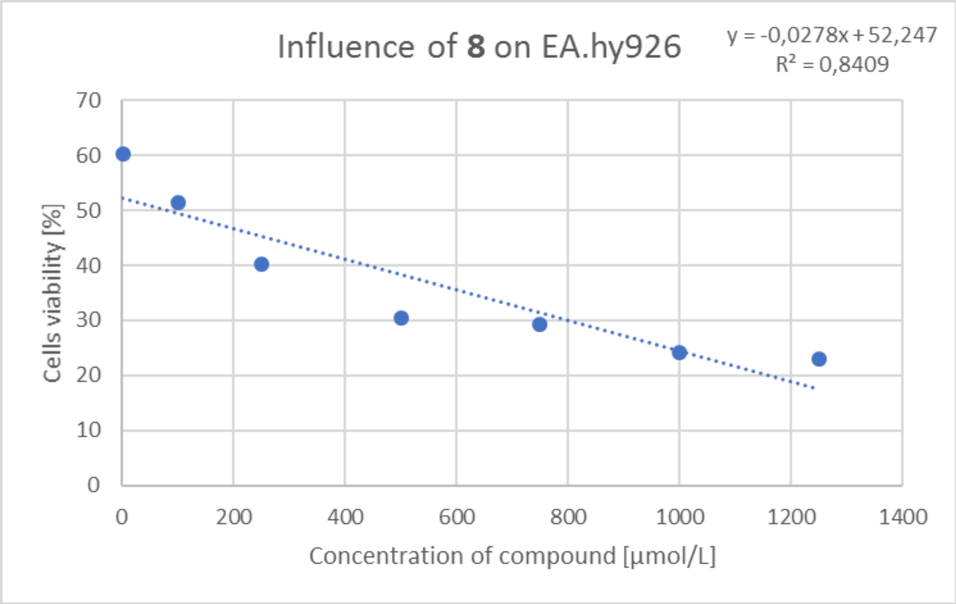
Influence of **8** on EA.hy926
y = -0,0278x + 52,247
R² = 0,8409
Cells viability [%]
Concentration of compound [µmol/L]
0
10
20
30
40
50
60
70
0
200
400
600
800
1000
1200
1400

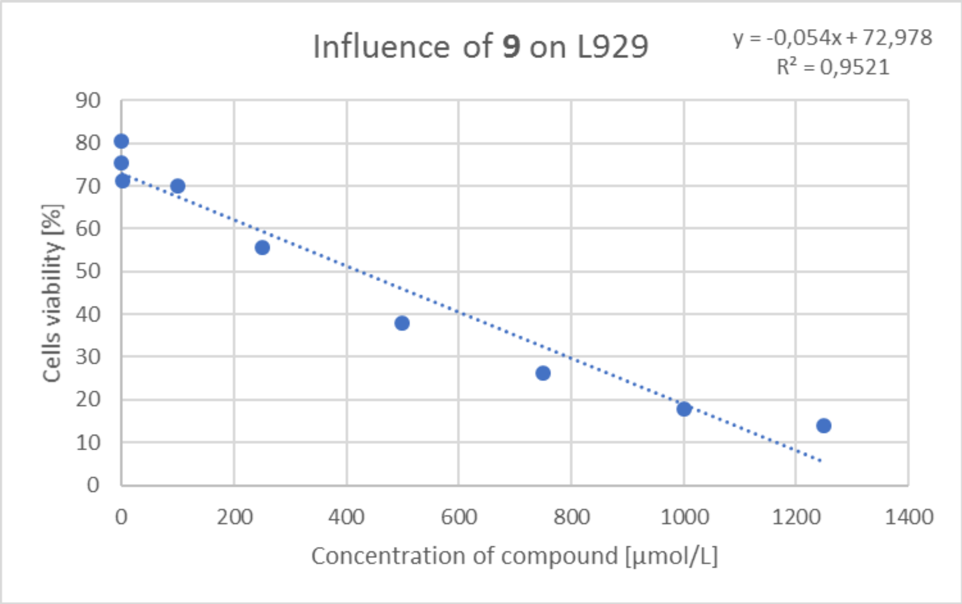
Influence of **9** on L929
y = -0,054x + 72,978
R² = 0,9521
Cells viability [%]
Concentration of compound [µmol/L]
0
10
20
30
40
50
60
70
80
90
0
200
400
600
800
1000
1200
1400

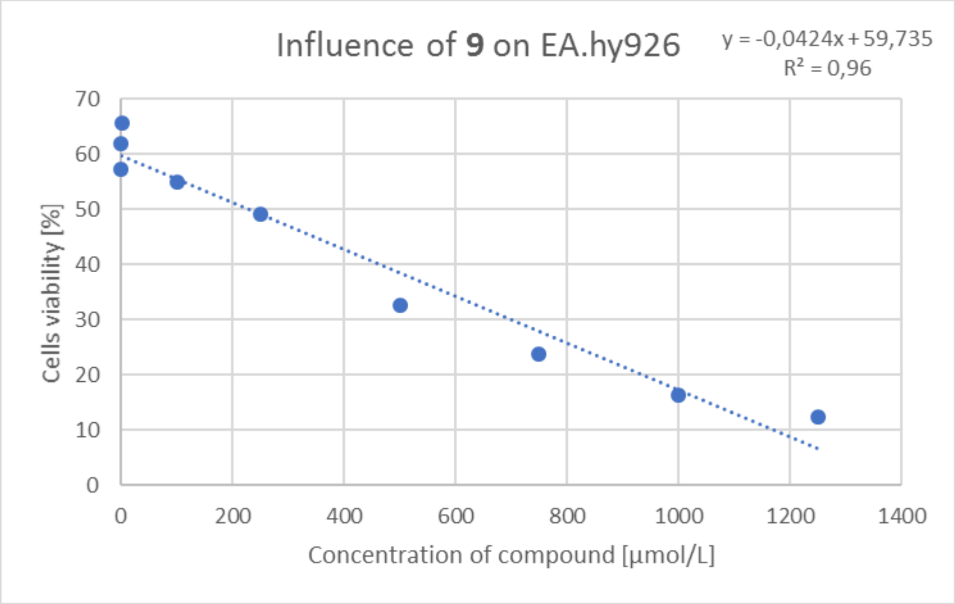
Influence of 9 on EA.hy926
y = -0,0424x + 59,735
R² = 0,96
Cells viability [%]
0
10
20
30
40
50
60
70
0
200
400
600
800
1000
1200
1400
Concentration of compound [µmol/L]

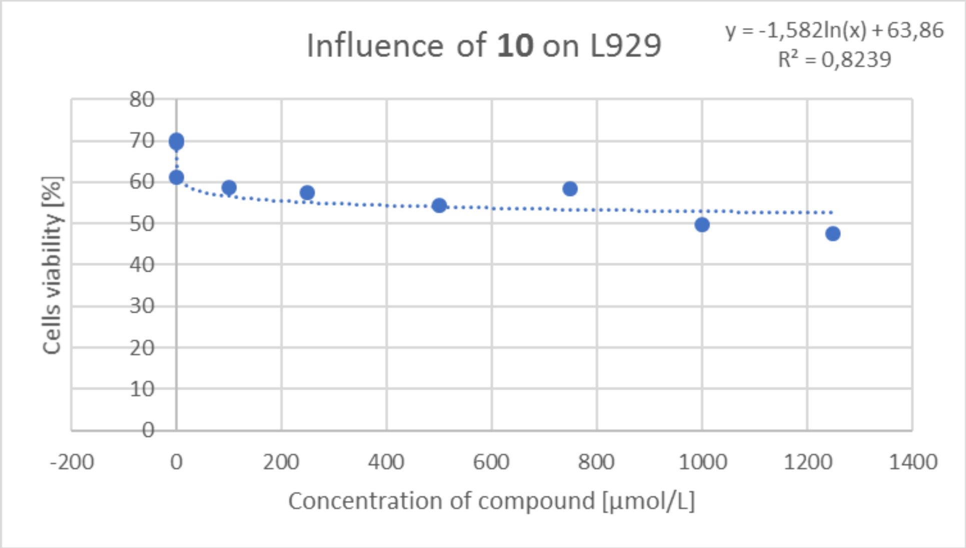
Influence of 10 on L929
y = -1,582ln(x) + 63,86
R² = 0,8239
Cells viability [%]
0
10
20
30
40
50
60
70
80
-200
0
200
400
600
800
1000
1200
1400
Concentration of compound [µmol/L]

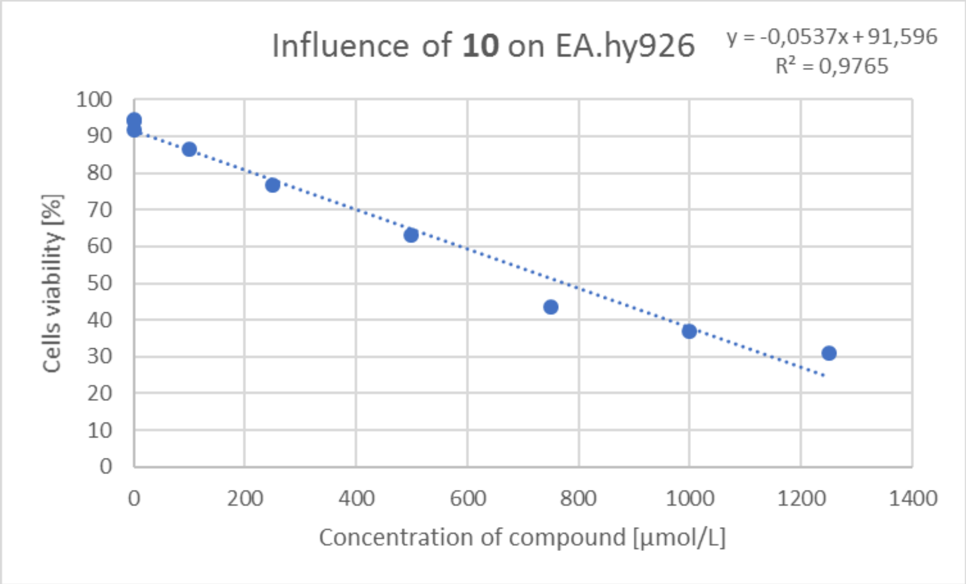
Influence of 10 on EA.hy926
y = -0,0537x + 91,596
R² = 0,9765
Cells viability [%]
0
10
20
30
40
50
60
70
80
90
100
0
200
400
600
800
1000
1200
1400
Concentration of compound [µmol/L]

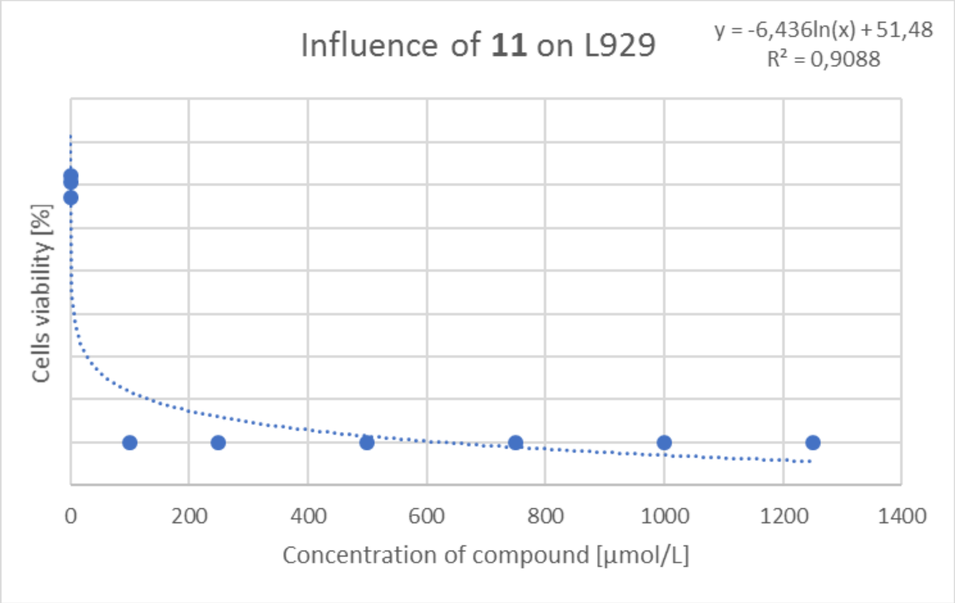
Influence of 11 on L929
y = -6,436ln(x) + 51,48
R² = 0,9088
Cells viability [%]
0
200
400
600
800
1000
1200
1400
Concentration of compound [µmol/L]

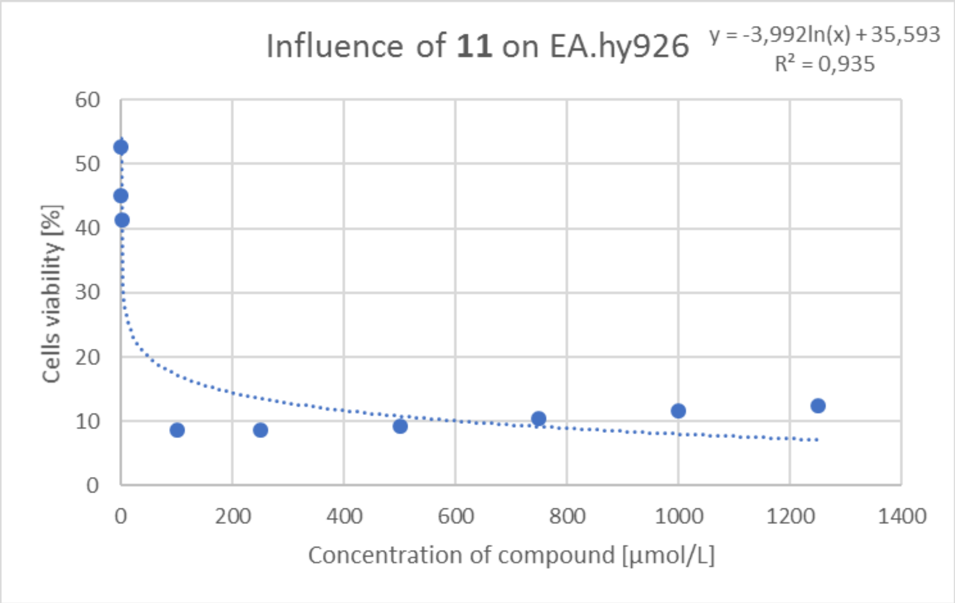
Influence of 11 on EA.hy926
y = -3,992ln(x) + 35,593
R² = 0,935
Cells viability [%]
60
50
40
30
20
10
0
0
200
400
600
800
1000
1200
1400
Concentration of compound [µmol/L]

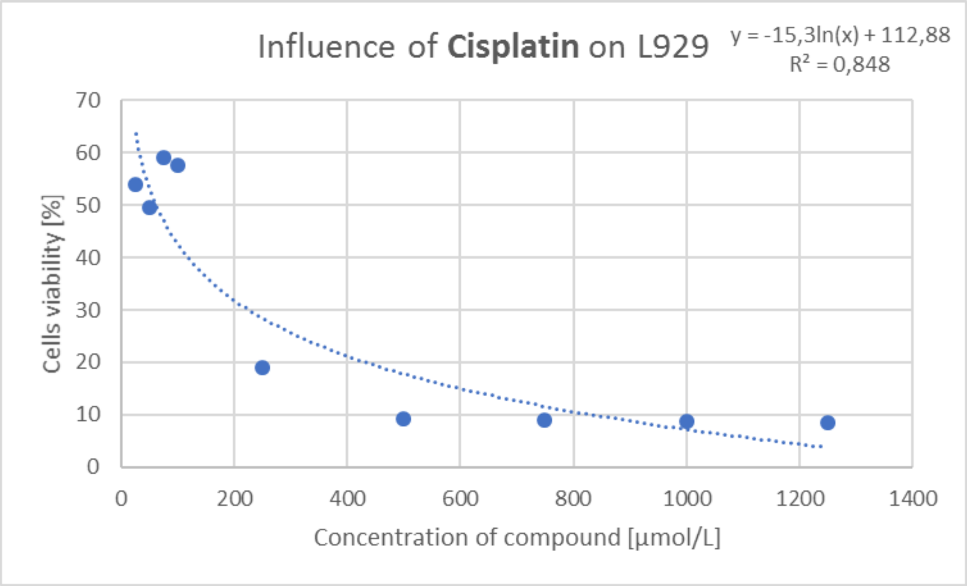
Influence of **Cisplatin** on L929
y = -15,3ln(x) + 112,88
R² = 0,848
Cells viability [%]
70
60
50
40
30
20
10
0
0
200
400
600
800
1000
1200
1400
Concentration of compound [µmol/L]

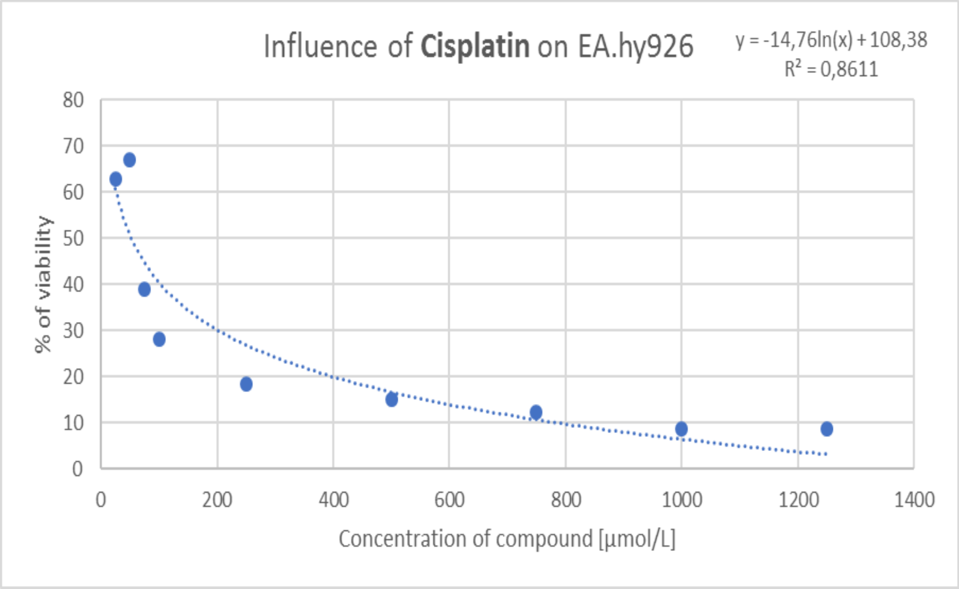
Influence of **Cisplatin** on EA.hy926
y = -14,76ln(x) + 108,38
R² = 0,8611
% of viability
80
70
60
50
40
30
20
10
0
0
200
400
600
800
1000
1200
1400
Concentration of compound [µmol/L]

Figure S4: ^{1}H NMR and ^{13}C NMR Spectra

^{1}H NMR Spectra (DMSO-d$_6$), **1**:

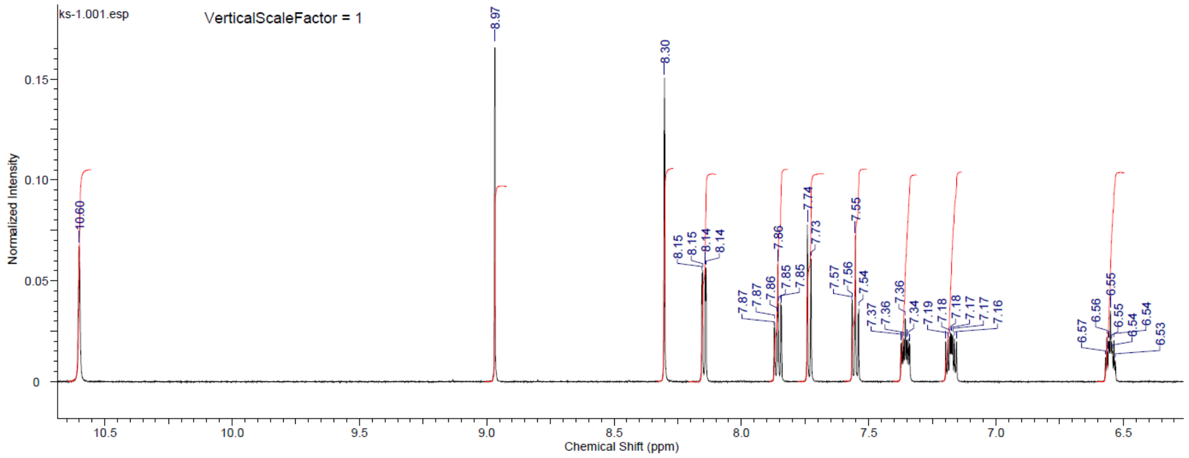

^{1}H NMR Spectra (DMSO-d$_6$), **3**:

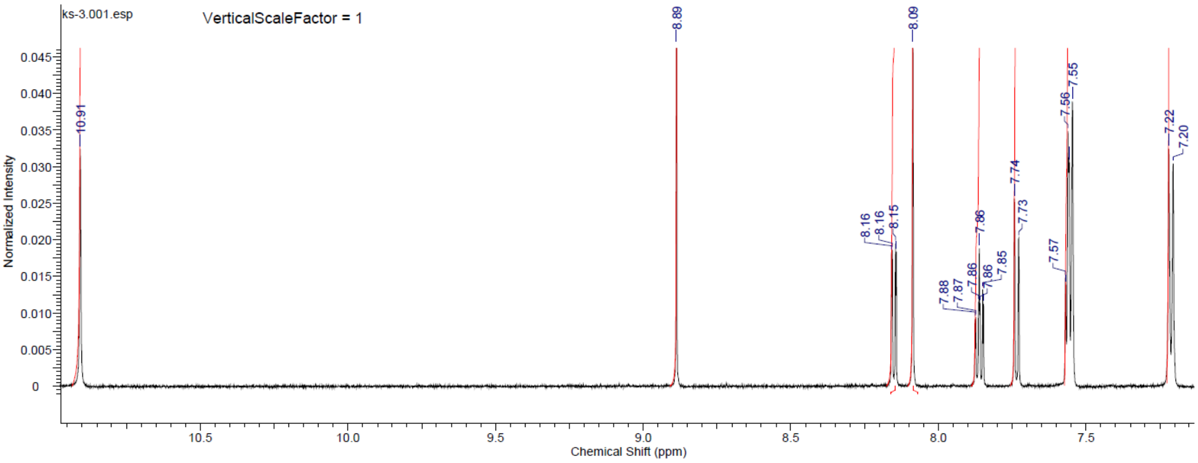

^{1}H NMR Spectra (DMSO-d$_6$), **4**:

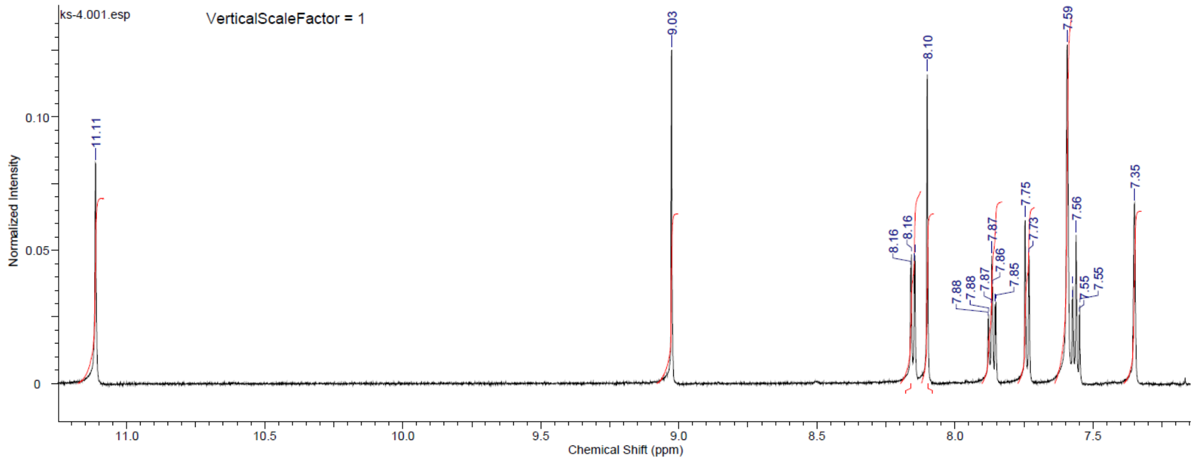

^{1}H NMR Spectra (DMSO-d$_6$), **5**:

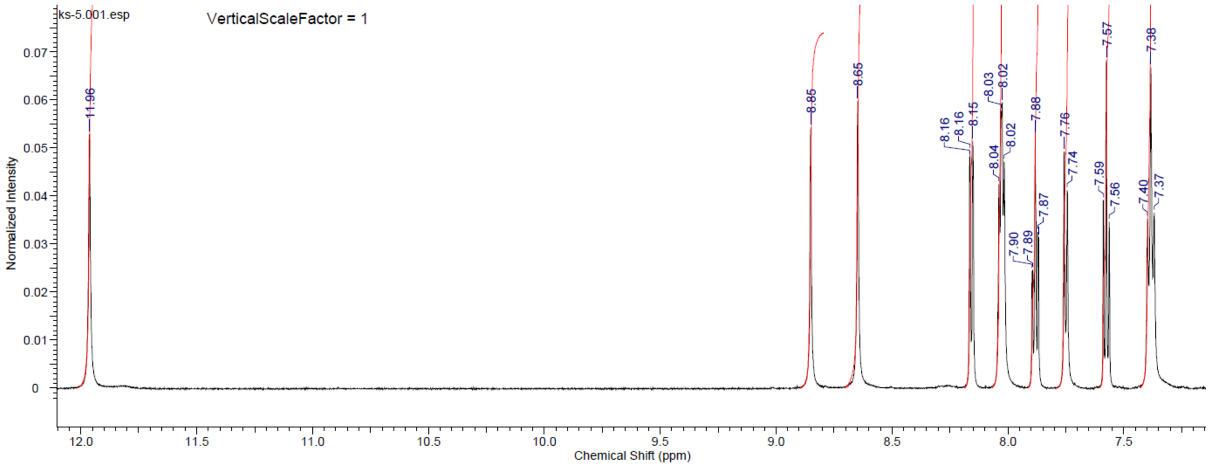

^{1}H NMR Spectra (DMSO-d$_6$), **6**:

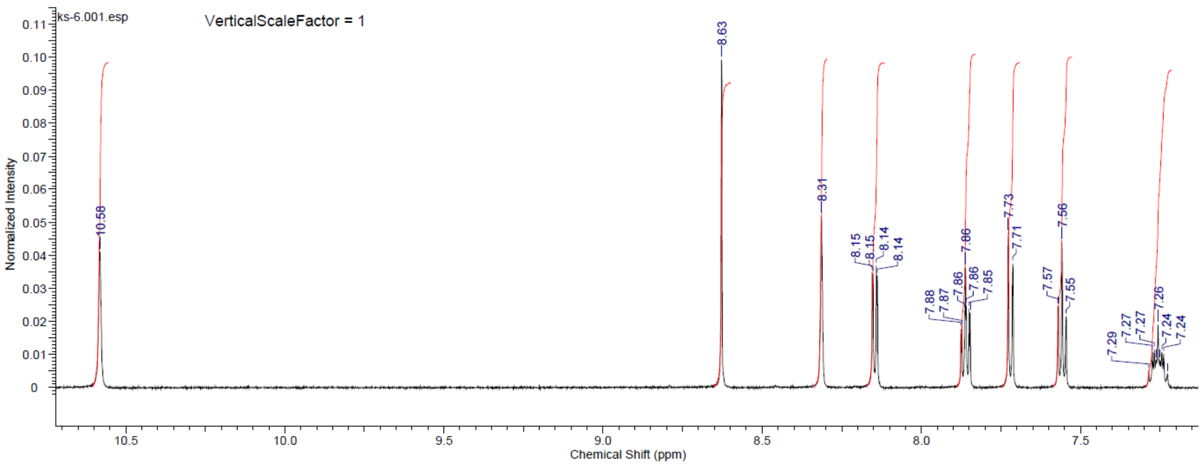

^{1}H NMR Spectra (DMSO-d$_6$), **7**:

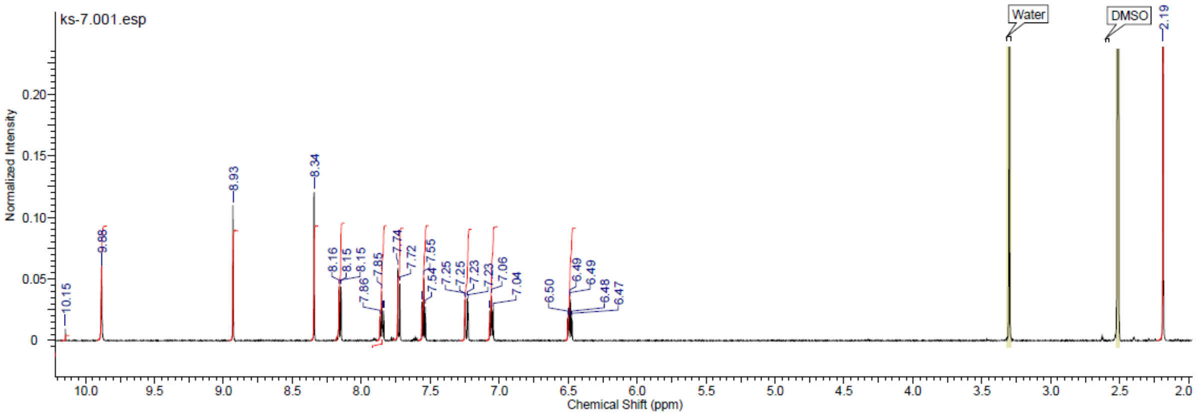

^{1}H NMR Spectra (DMSO-d$_6$), **8**:

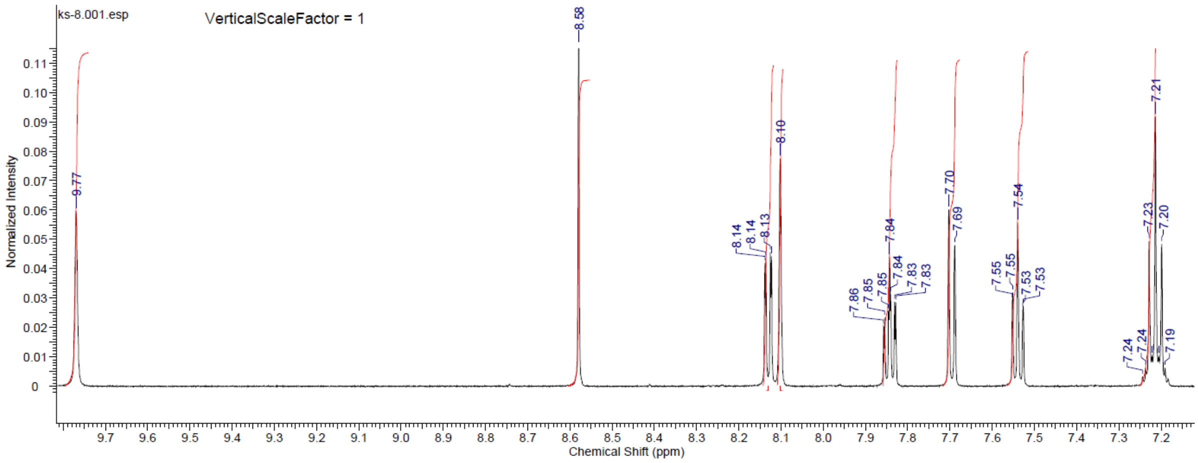

^{1}H NMR Spectra (DMSO-d$_6$), **9**:

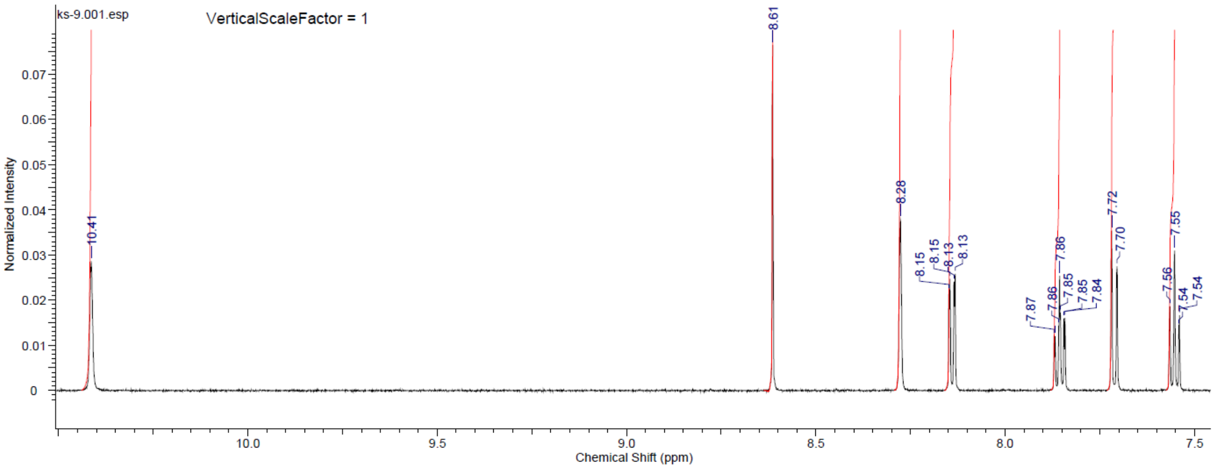

^{1}H NMR Spectra (DMSO-d$_6$), **10**:

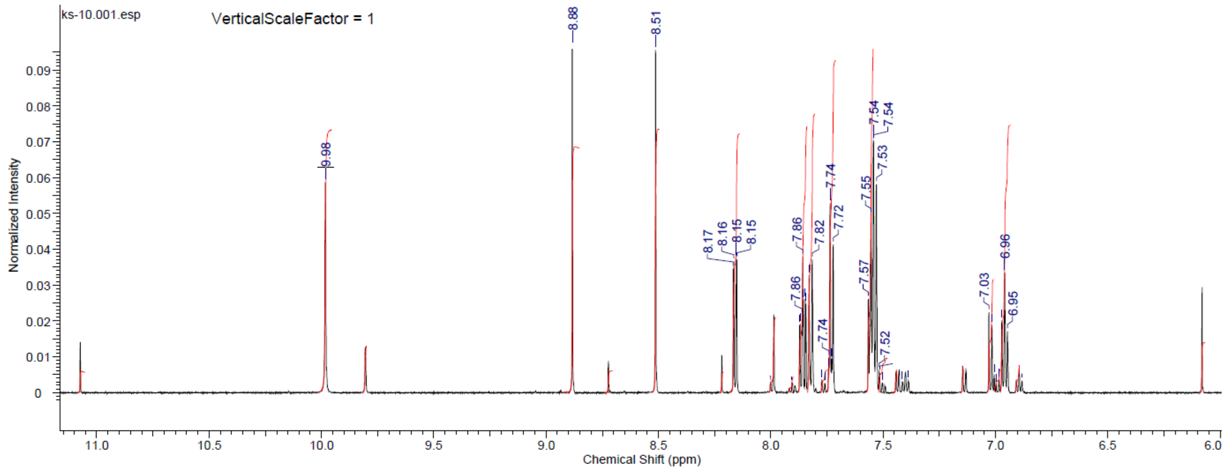

^{13}C NMR Spectra (DMSO-d6), **1**:

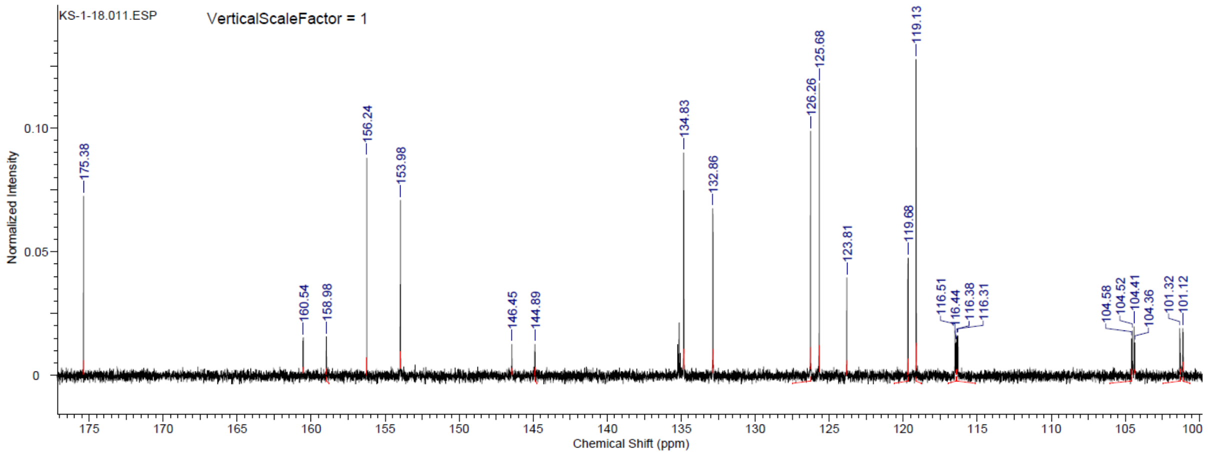

^{13}C NMR Spectra (DMSO-d6), **3**:

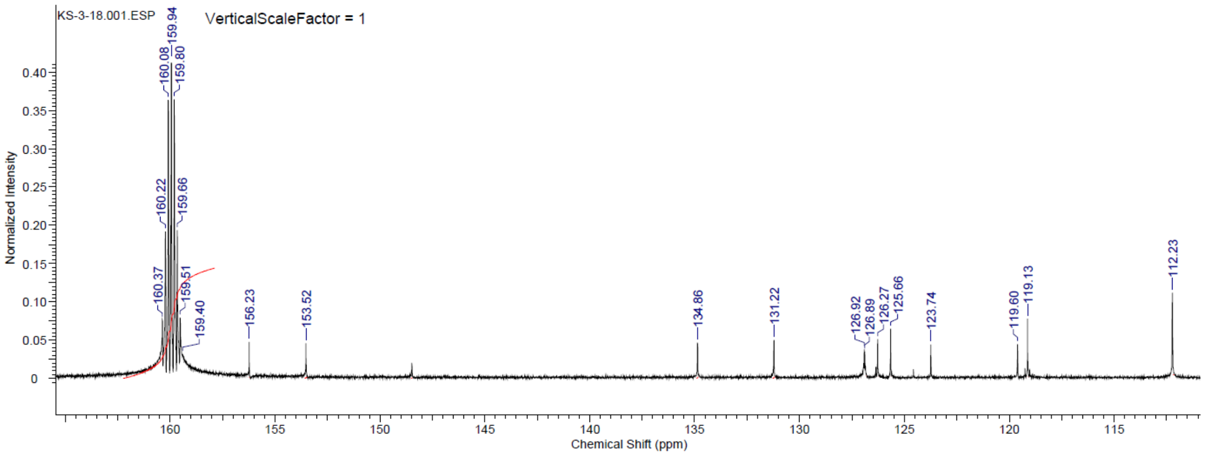

^{13}C NMR Spectra (DMSO-d6), **3**:

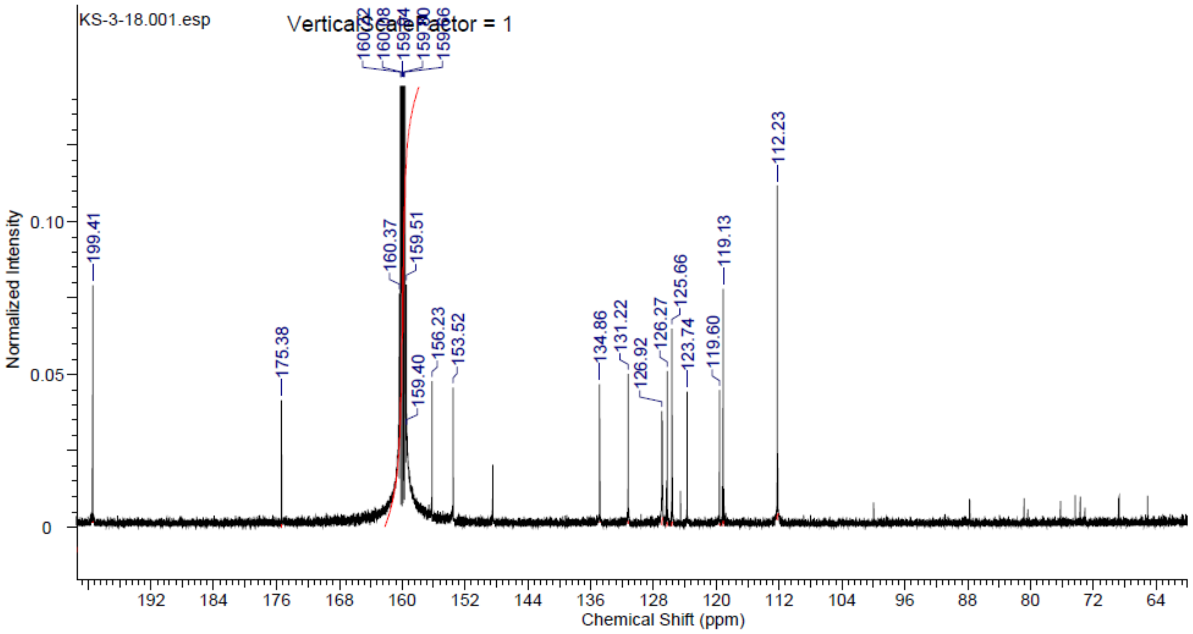

^{13}C NMR Spectra (DMSO-d_6), **5**:

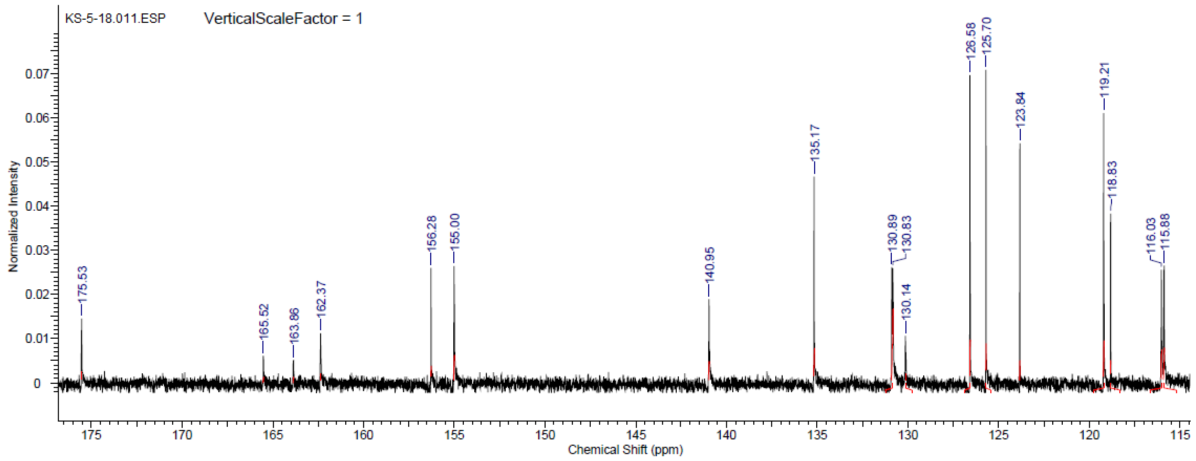

^{13}C NMR Spectra (DMSO-d_6), **6**:

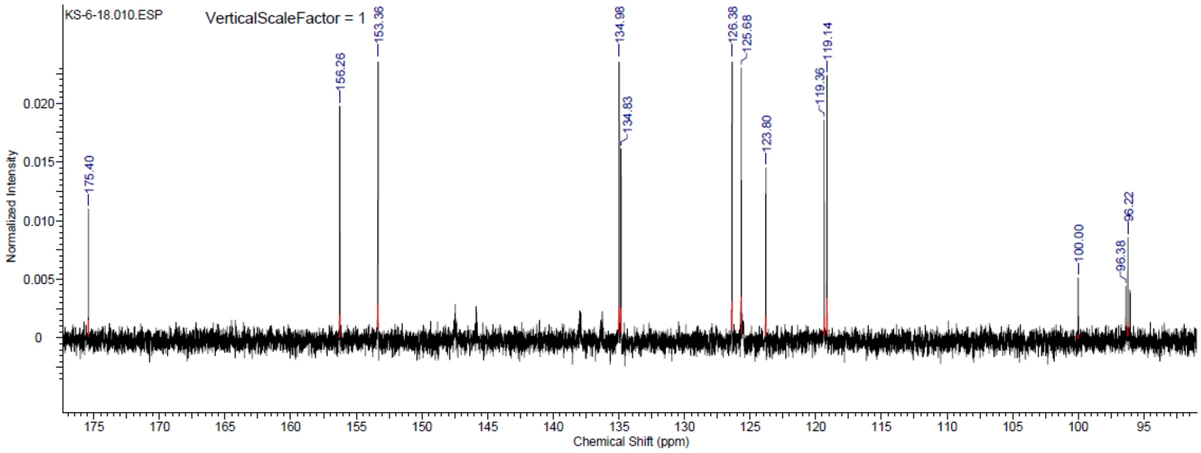

^{13}C NMR Spectra (DMSO-d_6), **7**:

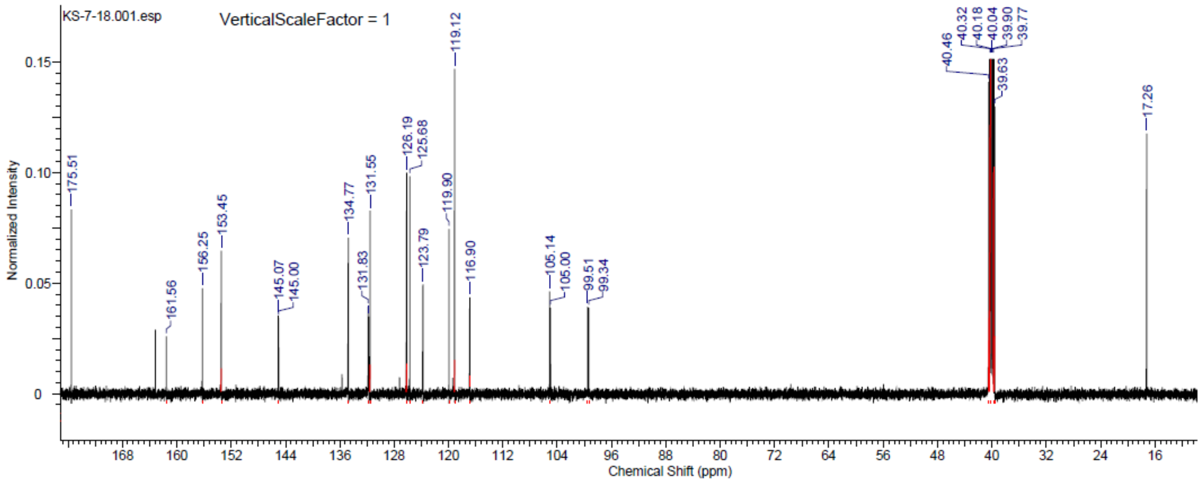

^{13}C NMR Spectra (DMSO-d_6), **8**:

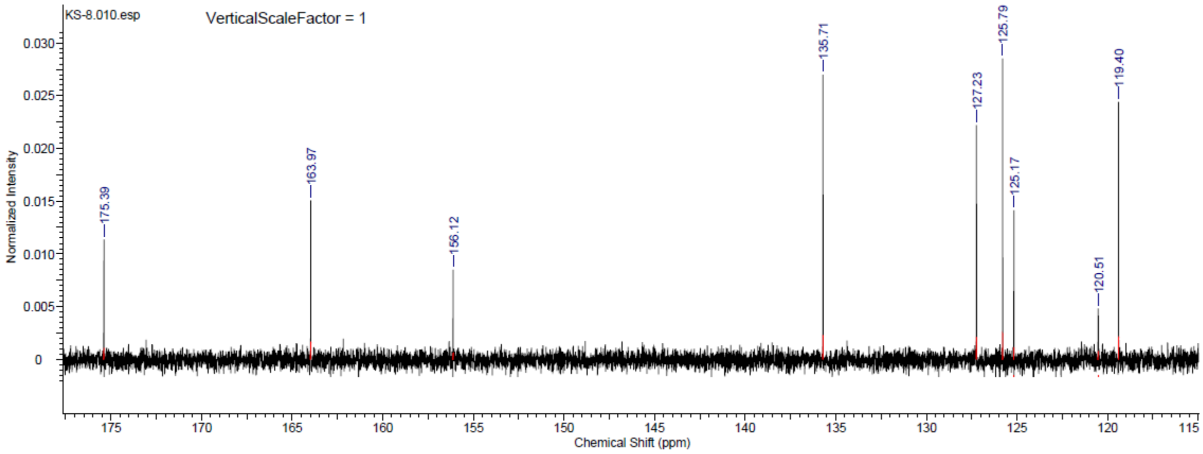

^{13}C NMR Spectra (DMSO-d_6), **9**:

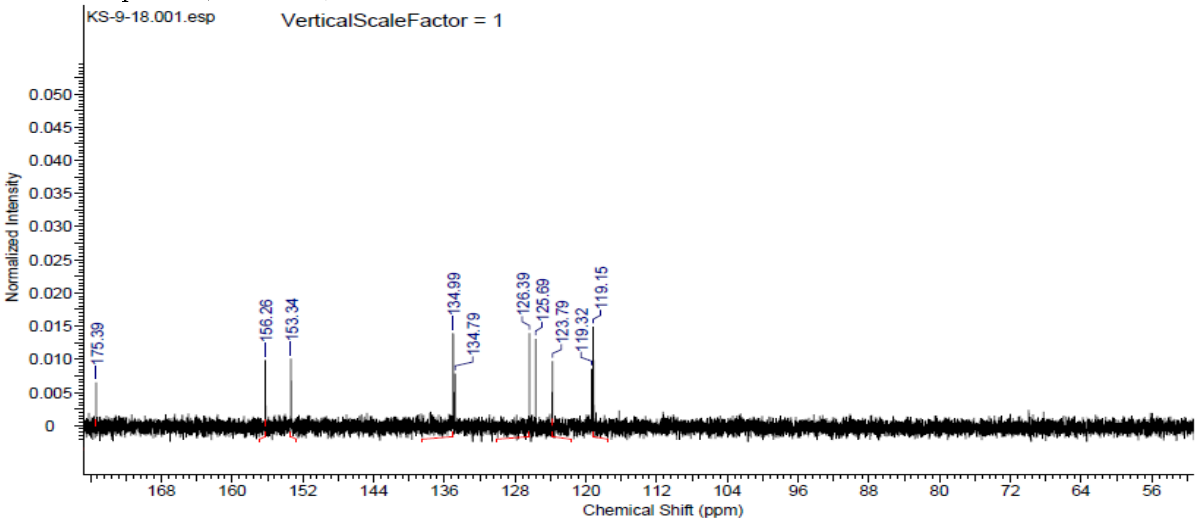

^{13}C NMR Spectra (DMSO-d_6), **10**:

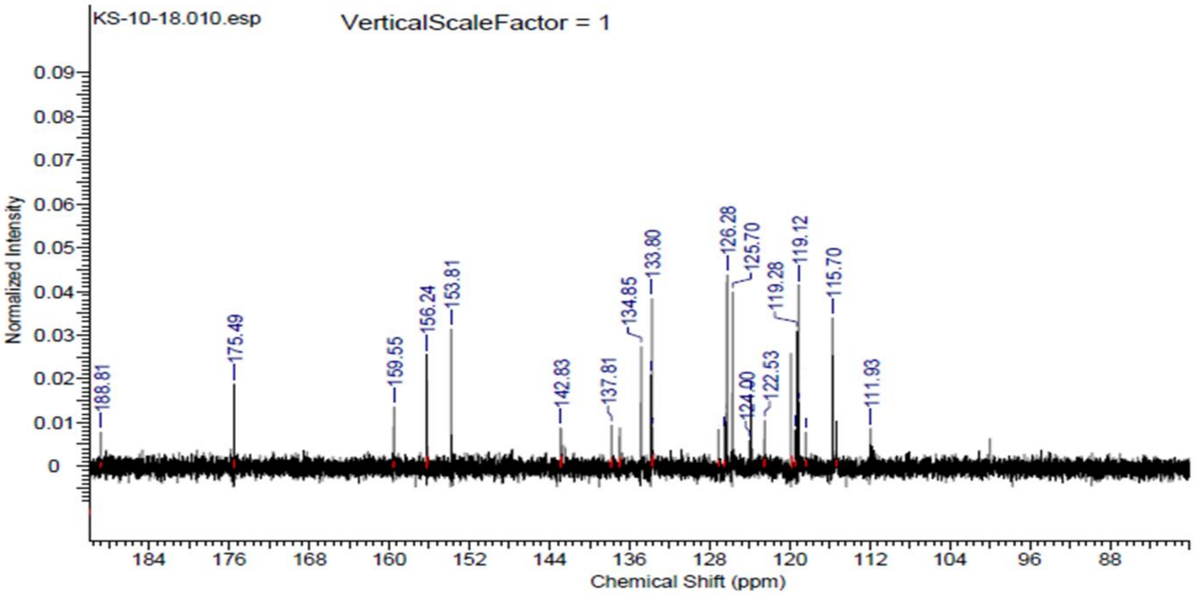

Supplement: Supplementary file 1 [file molecules-23-02067-s001.pdf]
